# Uterine morphology in normogonadotropic anovulation: a comparative study of polycystic ovary syndrome and hypothalamic-pituitary-ovarian dysfunction

**DOI:** 10.3389/fendo.2026.1781593

**Published:** 2026-04-02

**Authors:** Iwona Gawron, Karolina Zeman, Justyna Brodowicz, Robert Jach

**Affiliations:** 1 Chair of Gynecology and Obstetrics, Faculty of Medicine, Jagiellonian University Medical College, Krakow, Poland; 2 Clinical Department of Gynecological Endocrinology and Gynecological Oncology, University Hospital in Krakow, Krakow, Poland; 3 Chair of Clinical Biochemistry, Faculty of Medicine, Jagiellonian University Medical College, Krakow, Poland

**Keywords:** endometrium, hyperandrogenemia, hypothalamic-pituitary-ovarian axis dysfunction, myometrium, PCOS, uterine morphology

## Abstract

**Purpose:**

To compare uterine ultrasound measurements in women with normogonadotropic anovulation, specifically those with polycystic ovary syndrome (PCOS) and hypothalamic-pituitary-ovarian dysfunction (HPOD), with those of regularly menstruating women, and to assess the influence of clinical and biochemical parameters on these measurements.

**Methods:**

Uterine length, width, height, and volume, along with endometrial thickness and volume, measured using two- and three-dimensional transvaginal ultrasonography, were prospectively compared in women aged 18 to 45 from the aforementioned groups. Correlations between clinical parameters and uterine measurements, as well as between biochemical parameters and these measurements in anovulatory women, were analyzed.

**Results:**

Women with normogonadotropic anovulation had significantly reduced uterine and endometrial measurements compared to healthy women (all p ≤0.001). Women with PCOS showed significantly lower uterine length (p=0.045), height (p=0.004), and volume (p=0.009) than those with HPOD, with no significant endometrial differences. Among women with PCOS, those with hyperandrogenemia had thicker endometrium (p=0.036), with no significant differences in other measurements. All myometrial measurements significantly negatively correlated with anti-Müllerian hormone (AMH) and follicle-stimulating hormone (FSH), while positively correlating with estradiol and prolactin. Endometrial measurements negatively correlated with AMH and FSH concentrations, and positively with estradiol, prolactin, 17-hydroxyprogesterone, fasting insulin, and insulin resistance.

**Conclusions:**

A significant association was identified between menstrual cycle regularity and uterine morphology, influenced by hormonal, metabolic, and clinical factors. Estrogenic stimulation and metabolic status significantly affected uterine and endometrial dimensions, indicating that uterine morphology reflects the cumulative impact of reproductive and endocrine-metabolic influences, with important clinical implications for women with normogonadotropic anovulation.

## 1 Introduction

Ultrasonography, due to its widespread accessibility, reliability, and non-invasive nature, is the preferred imaging modality for evaluating pelvic structures. The assessment of uterine morphology, encompassing the measurement of its size and endometrial thickness, is crucial for determining its functional status and requires a comprehensive understanding of the anatomy and physiology of the reproductive system to accurately acquire and interpret ultrasonographic images (1, 2). Although a body of research has addressed the assessment of uterine normograms in specific populations and the analysis of measurement variations throughout the menstrual cycle (3) and over the lifespan (4), a substantial deficiency of data persists regarding the variability of uterine morphology in the context of normogonadotropic anovulation. The uterus serves as a target organ for steroid hormones and understanding uterine and endometrial morphology is essential for the diagnosis and treatment of various reproductive health conditions. The ultrasonographic assessment of the morphology of the uterine corpus is essential for the diagnosis of abnormal uterine bleeding, dysmenorrhea, pregnancy complications, and infertility. Furthermore, the visual evaluation of the endometrium is particularly relevant for diagnosing menstrual irregularities and for identifying structural alterations that may underlie abnormal uterine bleeding. The dimensions and volume of the myometrium and endometrium can influence clinical pregnancy rates in women undergoing assisted reproductive techniques, with both excessively small and large measurements potentially decreasing this rate. Inadequate dimensions may result from hypoestrogenism, while enlarged dimensions can arise from estrogen-progesterone imbalances associated with polycystic ovary syndrome (PCOS), increased uterine vascularization, or estrogen-dependent myometrial conditions such as adenomyosis. Additionally, both excessively thin and thick cystic endometrium can compromise endometrial receptivity and adversely affect embryo implantation. Despite established ultrasonographic criteria for ovarian assessment in PCOS (5), there is limited data regarding the myometrium and endometrium in women with PCOS and hypothalamic-pituitary-ovarian axis dysfunction (HPOD) (6). Moreover, considerable attention has been devoted to investigating complications specific to PCOS, such as insulin resistance, hyperinsulinemia, hyperandrogenemia, and systemic inflammation, which are known to act synergistically to impair endometrial function, leading to menstrual irregularities, infertility, and obstetric failure (7). However, the impact of these complications on the ultrasonographic morphology of the myometrium and endometrium in the context of normogonadotropic anovulation, as well as the differences between PCOS and HPOD, have not yet been studied. This study aimed to conduct a comparative ultrasonographic assessment of myometrial and endometrial measurements among women with PCOS and HPOD, while also evaluating the influence of selected clinical and biochemical variables on these measurements.

## 2 Materials and methods

A prospective cohort study was conducted among women diagnosed with menstrual irregularities from January 5, 2024, to December 31, 2024, in units affiliated with the Clinical Department of Gynecological Endocrinology and Gynecological Oncology at the University Hospital in Krakow. The study was approved by the Bioethics Committee of Jagiellonian University (no. 118.6120.53.2023) and conducted in accordance with the Helsinki Declaration, with informed written consent obtained from all participants. The study was registered in the ClinicalTrials.gov Protocol Registration and Results System (no. NCT06211608). The inclusion criteria were as follows: i) age 18 to 45 years, ii) no prior diagnosis and treatment for menstrual disorders or infertility. The exclusion criteria included: i) history of ovarian surgery, ii) use of medications that disrupt the hypothalamic-pituitary-ovarian axis, iii) presence of uterine tumors or congenital malformations. The control group consisted of regularly menstruating women of reproductive age, with non-pregnant and non-pathological uterus, reporting for preventive health care. All women participating in the study underwent a comprehensive physical examination, which included a medical interview and a gynecological evaluation featuring a vaginal speculum examination, bimanual examination, and pelvic ultrasound. Body Mass Index (BMI) was calculated using the formula: BMI= body weight [kg]/height [m]². Excess body hair was assessed using the modified Ferriman-Gallwey scale (mFG) (5). Based on the obtained data, a characterization of the population was conducted regarding demographic information (age, body weight, BMI), gynecological aspects (cycle length, bleeding pattern, menstrual-related pain, and infertility), obstetric history (number of pregnancies, deliveries, and miscarriages), ultrasonographic measurements, and, for women with irregular menstruation, biochemical parameters, along with an assessment of the correlations between these variables and the measured outcomes.

### 2.1 Ultrasonography of the reproductive organs

**Figure 1:**
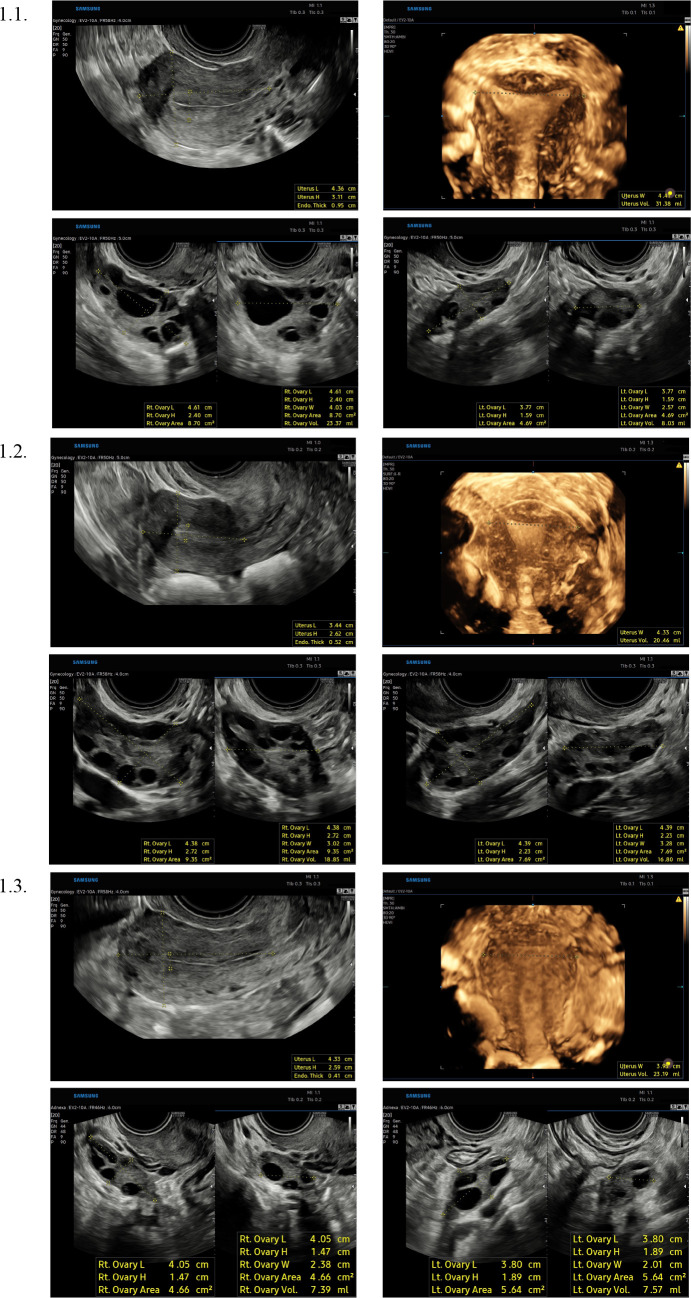
Ultrasonographic images of the female reproductive organs representative of women with regular menstrual cycles (healthy controls) (1.1), polycystic ovary syndrome (1.2), and hypothalamic-pituitary-ovarian axis dysfunction (1.3), accompanied by an illustration of the measurement techniques employed.

Two-dimensional and three-dimensional ultrasound imaging of the female reproductive organs was performed using the Samsung HERA W9 ultrasound device, employing transvaginal (EV2-10A) volume transducer (Samsung Electronics, Republic of Korea). The transvaginal examination was conducted with an emptied bladder, in a dorsal lithotomy position. The examination protocol included the evaluation of the cervix, uterine body, adnexa, and Douglas pouch. Uterine morphology was assessed in three planes: sagittal, transverse and coronal, achieved through HDVI™ volume rendering technology (Samsung Electronics, Republic of Korea). Volume acquisition was conducted using a standardized method (8), maintaining a maximum sweep angle of 180° after obtaining a sagittal section of the uterus, with the ultrasound beam oriented at approximately a 90° angle to the uterine axis. The ultrasound transducer was held stationary, and the examiner instructed the participant to remain still. The length of the uterine body was measured from the apex of the fundus to the internal cervical os, while the height was assessed in the anteroposterior dimension at the level of the fundus, both on the sagittal section. The width was measured at the fundal level using a coronal section obtained through 3D reconstruction. The volume was calculated using an automatic function based on the formula for an ellipsoid, accounting for three dimensions: length, width, and height of the organ, employing the simplified formula: V = (π/6) × length × width × height (in cm and reported in ml). The detection of at least 20 follicles ranging from 2 to 9 mm in diameter, along with the absence of a corpus luteum, dominant follicle, or functional cyst, was indicative of polycystic ovarian morphology (PCOM) (5). Ultrasonographic representations of the female reproductive organs in women with regular menstrual cycles (healthy controls), polycystic ovary syndrome, and hypothalamic-pituitary-ovarian axis dysfunction, along with an illustration of the measurement techniques, were presented in **Figure 1**. Uterine malformations identified through ultrasound were classified according to the European Society for Gynaecological Endoscopy (ESGE) consensus (9), with women presenting such anomalies excluded from the study. Ultrasound examinations were performed exclusively by two designated investigators (I.G., K.Z.) collaborating closely to ensure consistency in conducting and reporting measurements, thereby minimizing variability and enhancing the reliability of the collected data.

### 2.2 Biochemical analysis of blood plasma

Concentrations of specific biochemical parameters were determined using an automated Roche Cobas PRO/e801 analyzer (Roche Diagnostics, Basel, Switzerland) on a 10 ml venous blood sample collected after a minimum eight-hour overnight fast. The concentrations of serum follicle-stimulating hormone (FSH) [mIU/ml], luteinizing hormone (LH) [mIU/ml], prolactin (PRL) [μIU/ml], Anti-Müllerian hormone (AMH) [pmol/l], estradiol [pmol/l], testosterone [nmol/l], sex hormone-binding globulin (SHBG) [nmol/l], dehydroepiandrosterone sulfate (DHEA-S) [μmol/l], fasting insulin (FI) [μU/ml], and those measured at the 120-minute mark during the 75 g glucose tolerance test (120’75OGTTI) [μU/ml], along with thyroid-stimulating hormone (TSH) [μIU/ml], free triiodothyronine (fT3) [pmol/l], free thyroxine (fT4) [pmol/l], thyroid peroxidase antibodies (TPOAb) [IU/ml], thyroglobulin antibodies (TGAb) [IU/ml], and 17-hydroxyprogesterone (17-OHP) [ng/ml] were quantified utilizing electrochemiluminescence immunoassay (ECLIA). Fasting glucose (FG) [mmol/l] and glucose levels at 120 minutes during the 75 g glucose tolerance test (120’75OGTTG) [mmol/l], triglycerides (TG) [mmol/l], and total cholesterol (TC) [mmol/l] were assessed using enzymatic methods, while high-density lipoprotein cholesterol (HDL) [mmol/l] was evaluated spectrophotometrically. C-reactive protein (CRP) [mg/l] was analyzed using an immunoturbidimetric method. Low-density lipoprotein (LDL) cholesterol [mmol/l] was calculated using the Friedewald formula (10). The Homeostasis Model Assessment for insulin resistance (HOMA-IR) was determined by multiplying FI by FG and dividing by 22.5. The free androgen index (FAI) was calculated by dividing total testosterone concentration by SHBG concentration. Hyperandrogenemia was defined as a testosterone concentration greater than 1.67 nmol/L or a FAI greater than 5.

### 2.3 Statistical analysis

Quantitative variables were analyzed using descriptive statistics, which included the mean, standard deviation, median, quartiles, and range. Qualitative variables were evaluated by calculating the absolute and percentage frequencies of all possible values. The distributions of the variables were examined using the Kolmogorov-Smirnov test. The assessment of quantitative variable values between two groups was conducted using the Mann-Whitney U test. To examine quantitative variable values among three or more groups, the Kruskal-Wallis test was employed, followed by Dunn’s *post-hoc* test where statistically significant differences among groups were identified. Comparative analysis of qualitative variables across different groups was performed using the chi-square test (with Yates’ continuity correction for 2x2 contingency tables) or Fisher’s exact test in cases where the assumptions for the chi-square test regarding expected frequencies were not met. Correlations among quantitative variables were analyzed using Spearman’s rank correlation coefficient. A significance threshold of 0.05 was established, indicating that all p-values below 0.05 were considered indicative of statistically significant associations. The analyses were conducted using R software, version 4.5.1 (11). Assuming a 95% confidence level, a 5% margin of error, and a 50% population proportion, the normogonadotropic anovulation arm required a minimum sample size of 285 participants from a cohort of 1100 women undergoing diagnostic evaluation of menstrual irregularities. The healthy control group, based on 200 women undergoing routine examinations, required a minimum sample size of 132 participants.

## 3 Results

The study consecutively enrolled 648 women, including 452 with irregular menstrual cycles in the study arm and 196 with regular cycles in the control arm. The developed database has been made publicly available https://doi.org/10.7910/DVN/HZNRLK.

A comparative analysis of selected clinical parameters and ultrasonographic measurements of the uterus and ovarian characteristics in both study arms was presented in **Table 1**. Women with irregular cycles were significantly younger (p<0.001), scored higher on the mFG scale (p=0.008), exhibited a higher BMI (p=0.001), had a longer average cycle length (p<0.001), and lower percentage of pregnancies (p=0.025) and miscarriages (p=0.001). No significant intergroup differences were identified in the prevalence of dysmenorrhea, AUB, or history of childbirth (all p>0.05). All myometrial measurements in women with irregular menstrual cycles were significantly reduced, including length (p=0.001), height (p<0.001), width (p<0.001), and consequently the volume of the uterine corpus (p<0.001), as well as thickness (p<0.001) and volume of the endometrium (p<0.001). Ultrasonographic ovarian volume (p=0.016) and the prevalence of PCOM (p<0.001) were greater among women with irregular menstrual cycles. The frequencies of other ovarian findings, such as dominant follicle, simple cyst, and ovarian lesion, were not significantly different (all p-values >0.05).

**Table 1:** Comparative analysis of selected clinical variables between the study arms of women with regular and irregular menstrual cycles.

Variable		Irregular cycles (N = 452)	Regular cycles (N = 196)	Total (N = 648)	p
Age [years]	Mean (SD)	26.82 (5.14)	28.73 (5.72)	27.4 (5.39)	p<0.001 *
	Median (quartiles)	26 (23-30)	28 (24.75-33)	27 (24-31)	
	Range	18-44	18-45	18-45	
mFerriman - Gallwey scale [n]	Mean (SD)	5.19 (4.34)	4.37 (4.23)	4.94 (4.32)	p=0.008 *
	Median (quartiles)	4 (2-7)	3 (2-6)	4 (2-7)	
	Range	0-25	0-26	0-26	
BMI [kg/m²]	Mean (SD)	25.31 (6.11)	23.79 (5.44)	24.85 (5.96)	p=0.001 *
	Median (quartiles)	23.8 (20.7-29)	22.31 (19.96-25.11)	23.05 (20.43-28.01)	
	Range	15.94-50.94	16.6-46.07	15.94-50.94	
Average cycle length [days]	Mean (SD)	75.31 (77.26)	29.91 (5.18)	61.58 (67.86)	p<0.001 *
	Median (quartiles)	45 (36.75-75)	29 (28-30)	38 (30-60)	
	Range	15-540	21-71	15-540	
Pregnancies [n]	None	401 (88.72%)	157 (80.10%)	558 (86.11%)	p=0.025 *
	1 pregnancy	20 (4.42%)	19 (9.69%)	39 (6.02%)	
	2 pregnancies	20 (4.42%)	13 (6.63%)	33 (5.09%)	
	3 pregnancies	9 (1.99%)	4 (2.04%)	13 (2.01%)	
	4 pregnancies	2 (0.44%)	2 (1.02%)	4 (0.62%)	
	5 pregnancies	0 (0.00%)	1 (0.51%)	1 (0.15%)	
Childbirths [n]	None	411 (90.93%)	174 (88.78%)	585 (90.28%)	p=0.696
	1 childbirth	19 (4.20%)	12 (6.12%)	31 (4.78%)	
	2 childbirths	20 (4.42%)	9 (4.59%)	29 (4.48%)	
	3 childbirths	2 (0.44%)	1 (0.51%)	3 (0.46%)	
Miscarriages [n]	None	433 (95.80%)	175 (89.29%)	608 (93.83%)	p=0.001 *
	1 miscarriage	13 (2.88%)	9 (4.59%)	22 (3.40%)	
	2 miscarriages	2 (0.44%)	9 (4.59%)	11 (1.70%)	
	3 miscarriages	2 (0.44%)	3 (1.53%)	5 (0.77%)	
	4 miscarriages	2 (0.44%)	0 (0.00%)	2 (0.31%)	
Dysmenorrhea	No	273 (60.40%)	110 (56.12%)	383 (59.10%)	p=0.352
	Yes	179 (39.60%)	86 (43.88%)	265 (40.90%)	
Abnormal Uterine bleeding	No	321 (71.02%)	123 (62.76%)	444 (68.52%)	p=0.109
	HMB	115 (25.44%)	63 (32.14%)	178 (27.47%)	
	IMB	16 (3.54%)	10 (5.10%)	26 (4.01%)	
Endometrium- Thickness [cm]	Mean (SD)	0.73 (0.34)	0.86 (0.32)	0.77 (0.34)	p<0.001 *
	Median (quartiles)	0.68 (0.48-0.92)	0.88 (0.62-1.04)	0.73 (0.51-1)	
	Range	0.1-2.1	0.15-1.8	0.1-2.1	
Endometrium- Volume [ml]	Mean (SD)	2.96 (2.31)	3.82 (2.31)	3.22 (2.34)	p<0.001 *
	Median (quartiles)	2.3 (1.37-3.94)	3.42 (2.06-5.02)	2.56 (1.57-4.33)	
	Range	0.12-16.28	0.22-13.39	0.12-16.28	
Uterus- Length [cm]	Mean (SD)	4.31 (0.73)	4.5 (0.64)	4.37 (0.71)	p=0.001 *
	Median (quartiles)	4.24 (3.83-4.74)	4.42 (4.08-4.97)	4.3 (3.91-4.81)	
	Range	1.87-6.94	2.69-6.19	1.87-6.94	
Uterus- Height [cm]	Mean (SD)	3.36 (0.66)	3.61 (0.63)	3.44 (0.66)	p<0.001 *
	Median (quartiles)	3.29 (2.89-3.72)	3.55 (3.19-3.96)	3.41 (2.96-3.81)	
	Range	1.75-5.63	1.07-5.76	1.07-5.76	
Uterus- Width [cm]	Mean (SD)	4.56 (0.7)	4.82 (0.7)	4.64 (0.71)	p<0.001 *
	Median (quartiles)	4.57 (4.11-4.99)	4.81 (4.31-5.28)	4.62 (4.19-5.07)	
	Range	1.95-6.89	2.71-6.93	1.95-6.93	
Uterus- Volume [ml]	Mean (SD)	36.56 (17)	42.47 (16.84)	38.35 (17.15)	p<0.001 *
	Median (quartiles)	33.44 (25.14-44.03)	38.94 (30.25-53.27)	35.27 (26.34-46.5)	
	Range	6.04-120.39	5.44-117.82	5.44-120.39	
Right Ovarian Volume [ml]	Mean (SD)	13.28 (8.52)	13.11 (12.9)	13.23 (10.04)	p=0.177
	Median (quartiles)	12.06 (8.59-15.23)	11.44 (7.72-15.99)	11.91 (8.34-15.29)	
	Range	0.71-103.3	1.62-168.1	0.71-168.1	
Left Ovarian Volume [ml]	Mean (SD)	11.18 (5.9)	10.18 (5.52)	10.88 (5.8)	p=0.016 *
	Median (quartiles)	10.3 (7.17-13.96)	9.32 (6.04-13.21)	10.08 (6.76-13.65)	
	Range	0.67-58.65	2.6-34.79	0.67-58.65	
Polycystic ovarian morphology	No	122 (26.99%)	115 (58.67%)	237 (36.57%)	p<0.001 *
	Yes	330 (73.01%)	81 (41.33%)	411 (63.43%)	
Dominant follicle	No	363 (80.31%)	146 (74.49%)	509 (78.55%)	p=0.12
	Yes	89 (19.69%)	50 (25.51%)	139 (21.45%)	
Simple Cyst	No	436 (96.46%)	186 (94.90%)	622 (95.99%)	p=0.476
	Yes	16 (3.54%)	10 (5.10%)	26 (4.01%)	
Ovarian Lesion	No	441 (97.57%)	186 (94.90%)	627 (96.76%)	p=0.128
	Yes	11 (2.43%)	10 (5.10%)	21 (3.24%)	

p, Qualitative variables, chi-squared or Fisher’s exact test; Quantitative variables: Mann-Whitney test, * statistically significant (p<0.05); SD, standard deviation; quartiles, lower quartile (Q1), upper quartile (Q3); BMI, body mass index.

The comparative analysis of uterine measurements and concentrations of selected biochemical parameters in women with irregular menstrual cycles diagnosed with PCOS, compared to those with HPOD, was presented in **Table 2**. Women with PCOS demonstrated significantly reduced uterine length (p=0.045), height (0.004), and volume (p=0.009) compared to women with HPOD. Endometrial measurements, however, did not reveal significant differences (all p-values >0.05). Significant differences in concentrations were observed for AMH, LH, testosterone, DHEA-S, fT3, FI, TG, and 17-OHP, which were higher in individuals with PCOS, while estradiol, SHBG, and vitamin D were lower (all p-values <0.05).

**Table 2:** Comparative analysis of uterine measurements in women with irregular menstrual cycles diagnosed with polycystic ovary syndrome (PCOS) compared to those diagnosed with hypothalamic-pituitary-ovarian axis dysfunction (HPOD).

Parameter		PCOS (N = 375)	HPOD (N = 77)	Total (N = 452)	p
Endometrium – Thickness [cm]	Mean ± SD	0.72 ± 0.34	0.77 ± 0.34	0.73 ± 0.34	0.197
	Median (quartiles)	0.68 (0.47-0.91)	0.71 (0.51-1.02)	0.68 (0.48-0.92)	
	Range	0.1-2.1	0.16-1.8	0.1-2.1	
Endometrium – volume [ml]	Mean ± SD	2.92 ± 2.38	3.12 ± 1.95	2.96 ± 2.31	0.074
	Median (quartiles)	2.11 (1.34-3.83)	2.61 (1.62-4.06)	2.3 (1.37-3.94)	
	Range	0.12-16.28	0.13-10.91	0.12-16.28	
Uterus – Length [cm]	Mean ± SD	4.28 ± 0.72	4.47 ± 0.79	4.31 ± 0.73	0.045*
	Median (quartiles)	4.21 (3.82-4.69)	4.41 (4-4.97)	4.24 (3.83-4.74)	
	Range	2.11-6.94	1.87-6.06	1.87-6.94	
Uterus – Height [cm]	Mean ± SD	3.32 ± 0.64	3.56 ± 0.72	3.36 ± 0.66	0.004*
	Median (quartiles)	3.26 (2.87-3.68)	3.49 (3.08-4.01)	3.29 (2.89-3.72	
	Range	1.75-5.63	2.11-5.6	1.75-5.63	
Uterus - Width [cm]	Mean ± SD	4.56 ± 0.7	4.59 ± 0.72	4.56 ± 0.7	0.698
	Median (quartiles)	4.56 (4.12-4.99)	4.58 (4.11-5.02)	4.57 (4.11-4.99)	
	Range	2.21-6.89	1.95-6.49	1.95-6.89	
Uterus- Volume [ml]	Mean ± SD	35.71 ± 16.57	40.71 ± 18.49	36.56 ± 17	0.009*
	Median (quartiles)	32.15 (24.5-43.01)	37.79 (29.25-49.64)	33.45 (25.14-44.04	
	Range	6.04-120.39	7.51-100.61	6.04 -120.39	
Anti-Müllerian hormone [pmol/l]	Mean ± SD	51.95 ± 35.66	19.63 ± 11.55	46.44 ± 35	<0.001*
	Median (quartiles)	44.2 (30.75-62.15)	17.8 (11.2-25.4)	40 (24.53-56.73)	
	Range	0.07-398	0.14-48	0.07-398	
FSH [mIU/ml]	Mean ± SD	5.73 ± 4.14	5.43 ± 2.65	5.68 ± 3.92	0.444
	Median (quartiles)	5.6 (4.12-6.79)	5.21 (3.43-6.88)	5.59 (4.05-6.82)	
	Range	1.08-60.6	1.26-13.8	1.08-60.6	
LH [mIU/ml]	Mean ± SD	13.09 ± 9.9	7.77 ± 6.86	12.19 ± 9.66	<0.001*
	Median (quartiles)	11.1 (6.31-17.1)	6.36 (3.9-9.67)	9.75 (5.83-15.83)	
	Range	0.3-70.2	1.53-45.6	0.3-70.2	
Estradiol [pmol/l]	Mean ± SD	343.2 ± 325.39	419.78 ± 338.46	356.24 ± 328.54	0.019*
	Median (quartiles)	220 (159-410.5)	307 (171-539)	229.5 (160-450.75)	
	Range	18-2532	65.1-1513	18-2532	
Prolactin [μIU/ml]	Mean ± SD	355.72 ± 331.85	356.42 ± 274.18	355.84 ± 322.48	0.623
	Median (quartiles)	303 (218.5- 423)	303 (201-408)	303 (213.75-421.5)	
	Range	49.6-5657	70.9-1925	49.6-5657	
Testosterone [nmol/l]	Mean ± SD	1.78 ± 0.73	1.07 ± 0.34	1.66 ± 0.73	<0.001*
	Median (quartiles)	1.74 (1.25; 2.21)	1.11 (0.85; 1.34)	1.56 (1.15-2.11)	
	Range	0.16-4.68	0.29-1.67	0.16-4.68	
SHBG [nmol/l]	Mean ± SD	51.12 ± 28.54	59.62 ± 25.23	52.57 ± 28.16	0.002*
	Median (quartiles)	45.8 (30.2-64.5)	53.7 (42.8-75.8)	47.8 (32.48-66.3)	
	Range	10.1-200	17.7-132	10.1-200	
FAI [n]	Mean ± SD	4.88 ± 4	2.1 ± 1.04	4.4 ± 3.82	<0.001*
	Median (quartiles)	3.63 (2.24-6.37)	1.85 (1.36-2.64)	3.18 (1.94-5.73)	
	Range	0.47-26.72	0.61-4.86	0.47-26.72	
DHEA-S [μmol/l]	Mean ± SD	8.83 ± 3.78	6.99 ± 2.98	8.52 ± 3.72	<0.001*
	Median (quartiles)	8.25 (6.23-10.55)	6.19 (4.88-8.72)	8.01 (5.94-10.1)	
	Range	2.14-21.2	1.64-18.4	1.64-21.2	
TSH [μIU/ml]	Mean ± SD	2.1 ± 1.21	2.19 ± 2.37	2.11 ± 1.47	0.218
	Median (quartiles)	1.87 (1.37-2.59)	1.73 (1.23-2.31)	1.85 (1.37-2.49)	
	Range	0.13-14.7	0.01-20.4	0.01-20.4	
fT3 [pmol/l]	Mean ± SD	5.45 ± 0.81	5.06 ± 1.08	5.38 ± 0.87	0.001*
	Median (quartiles)	5.5 (4.94-5.98)	5.08 (4.35-5.59)	5.43 (4.86-5.97)	
	Range	2.71-7.6	2.93-9.7	2.71-9.7	
fT4 [pmol/l]	Mean ± SD	16.41 ± 2.42	16.13 ± 2.87	16.36 ± 2.5	0.115
	Median (quartiles)	16.3 (14.9-17.9)	15.7 (14.3-17.6)	16.2 (14.7; 17.83)	
	Range	8.85-27.9	8.67-28.4	8.67-28.4	
anti-TPO [IU/ml]	Mean ± SD	25.53 ± 53.82	34.84 ± 83.03	27.11 ± 59.8	0.478
	Median (quartiles)	12.1 (9-16.2)	10.9 (9-15.7)	12 (9-16.15)	
	Range	9-470	9-502	9-502	
anti-TG [IU/ml]	Mean ± SD	52.89 ± 209.28	40.26 ± 72.14	50.74 ± 192.93	0.371
	Median (quartiles)	16.6 (15.1-18.9)	16.4 (14.8-18)	16.6 (15-18.8)	
	Range	11.5-3660	12.5-323	11.5-3660	
Fasting glucose [mmol/l]	Mean ± SD	4.84 ± 0.63	4.77 ± 0.36	4.83 ± 0.59	0.178
	Median (quartiles)	4.77 (4.51-5.04)	4.72 (4.51-4.92)	4.77 (4.51-5.02)	
	Range	3.66-11.2	4.06-5.82	3.66-11.2	
120’ OGTT Glucose [mmol/l]	Mean ± SD	5.37 ± 1.43	5.18 ± 1.09	5.34 ± 1.38	0.176
	Median (quartiles)	5.24 (4.4-6.18)	5.15 (4.52-5.84)	5.21 (4.41-6.11)	
	Range	2.13-12.2	2.48-7.85	2.13-12.2	
Fasting insulin [μU/ml]	Mean ± SD	11.62 ± 9.37	8.62 ± 4.93	11.11 ± 8.84	0.006*
	Median (quartiles)	8.98 (6.36-13.45)	6.9 (5.49-10.6)	8.69 (6.13-12.8)	
	Range	1.94-71.9	0.9-31.3	0.9-71.9	
120’ OGTT Insulin [μU/ml]	Mean ± SD	67.42 ± 53.13	54.14 ± 36.21	65.15 ± 50.86	0.064
	Median (quartiles)	52.2 (33.6-85.95)	45.1 (27.5-70)	49.95 (32.68-83.7)	
	Range	5.67-387	7.92-186	5.67-387	
HOMA-IR [n]	Mean ± SD	2.63 ± 2.68	1.85 ± 1.11	2.49 ± 2.5	0.008*
	Median (quartiles)	1.89 (1.28-2.95)	1.53 (1.1-2.22)	1.85 (1.23-2.82)	
	Range	0.37-24.16	0.19-6.12	0.19-24.16	
Total cholesterol [mmol/l]	Mean ± SD	4.62 ± 0.83	4.7 ± 0.8	4.64 ± 0.82	0.477
	Median (quartiles)	4.6 (4-5.1)	4.6 (4.2-5.1)	4.6 (4.1-5.1)	
	Range	2.4-7.6	2.9-8.4	2.4-8.4	
HDL cholesterol [mmol/l]	Mean ± SD	1.67 ± 0.4	1.77 ± 0.49	1.69 ± 0.42	0.094
	Median (quartiles)	1.64 (1.4-1.89)	1.71 (1.47-2.06)	1.64 (1.41-1.91)	
	Range	0.76-2.85	0.76-3.16	0.76-3.16	
LDL cholesterol [mmol/l]	Mean ± SD	2.52 ± 0.71	2.55 ± 0.71	2.52 ± 0.71	0.741
	Median (quartiles)	2.5 (2-3)	2.5 (2-3)	2.5 (2-3)	
	Range	0.7-6.1	1.2-5.2	0.7-6.1	
Triglycerides [mmol/l]	Mean ± SD	0.95 ± 0.52	0.81 ± 0.42	0.92 ± 0.51	0.017*
	Median (quartiles)	0.81 (0.61-1.1)	0.69 (0.54-1.01)	0.78 (0.61-1.09)	
	Range	0.27-4.29	0.32-2.94	0.27-4.29	
AST [IU/l]	Mean ± SD	23.36 ± 8.18	23.21 ± 10.54	23.34 ± 8.61	0.175
	Median (quartiles)	22 (19-25)	21 (17-24)	22 (19-25)	
	Range	9-94	12-90	9-94	
ALT [IU/l]	Mean ± SD	23.02 ± 13.49	20.97 ± 12.9	22.67 ± 13.4	0.050
	Median (quartiles)	19 (14.5-26)	17 (13-23)	19 (14-26)	
	Range	6-90	7-74	6-74	
CRP [mg/l]	Mean ± SD	2.19 ± 2.96	2.47 ± 3.38	2.23 ± 3.04	0.476
	Median (quartiles)	0.83 (0.6-2.55)	1.21 (0.6-2.32)	0.89 (0.6-2.54)	
	Range	0.6-20.3	0.6-15.4	0.6-20.3	
Vitamin D [ng/ml]	Mean ± SD	30.08 ± 12.22	35.19 ± 16	30.95 ± 13.07	0.033*
	Median (quartiles)	28.2 (22.35-35.7)	28.7 (24.8-40.4)	28.25 (22.68-36.7)	
	Range	9.18-102	14.2-77.6	9.18-102	
17-OH Progesterone [ng/ml]	Mean ± SD	1.43 ± 0.94	1.4 ± 1.3	1.43 ± 1.01	0.038*
	Median (quartiles)	1.16 (0.81-1.77)	0.96 (0.57-1.79)	1.12 (0.75-1.77)	
	Range	0.18-5.97	0.15-7.47	0.15-7.47	

p, Mann-Whitney test; * statistically significant (p<0.05); SD, standard deviation. quartiles: lower quartile (Q1)- upper quartile (Q3); PCOS, polycystic ovary syndrome; HPOD, hypothalamic-pituitary-ovarian axis dysfunction; AMH, anti-Müllerian hormone; FSH, follicle-stimulating hormone; LH, luteinizing hormone; SHBG, sex hormone binding globulin; FAI, free androgen index; DHEA-S, dehydroepiandrosterone sulfate; TSH, thyroid-stimulating hormone; fT3, free triiodothyronine; fT4, free thyroxine; TPOAb, thyroid peroxidase antibodies; TGAb, thyroglobulin antibodies; OGTT, 75-g oral glucose tolerance test; HOMA-IR, homeostasis model assessment for insulin resistance; HDL, high-density lipoprotein; LDL, low-density lipoprotein; AST, aspartate transaminase; ALT, alanine transaminase; CRP, C-reactive protein.

The comparative analysis of uterine measurements and concentrations of selected biochemical parameters in women diagnosed with PCOS with hyperandrogenemia and those without hyperandrogenemia was outlined in **Table 3**. Women with hyperandrogenemia exhibited significantly increased endometrial thickness compared to women without hyperandrogenemia (p=0.036). However, other uterine measurements did not show significant differences. Aside from testosterone, significant differences in concentrations were observed for AMH, FSH, LH, estradiol, PRL, DHEA-S, fT3, 120’OGTTG, FI, 120’OGTTI, AST, ALT, and 17-OHP, which were higher in hyperandrogenemia, while SHBG and HDL cholesterol were lower (all p-values <0.05).

**Table 3:** Comparative analysis of uterine measurements in women diagnosed with polycystic ovary syndrome (PCOS) with and without hyperandrogenemia.

Parameter		Hyperandrogenemia (n=230)	Normoandrogenemia (n=145)	Total (n=375)	p
Endometrium- Thickness [cm]	Mean ± SD	0.75 ± 0.33	0.68 ± 0.34	0.73 ± 0.34	0.036*
	Median (quartiles)	0.7 (0.51-0.9)	0.61 (0.41-0.91)	0.68 (0.47-0.91)	
	Range	0.1-2.1	0.1-2.01	0.1-2.1	
Endometrium- Volume [ml]	Mean ± SD	3 ± 2.46	2.8 ± 2.25	2.92 ± 2.38	0.294
	Median (quartiles)	2.19 (1.46-3.79)	2.05 (1.15-4.01)	2.11 (1.34-3.83)	
	Range	0.26-16.28	0.12-12.98	0.12-16.28	
Uterus- Length [cm]	Mean ± SD	4.25 ± 0.73	4.32 ± 0.7	4.28 ± 0.72	0.154
	Median (quartiles)	4.16 (3.78-4.64)	4.28 (3.89-4.72)	4.21 (3.82-4.69)	
	Range	2.11-6.94	2.28-6.55	2.11-6.94	
Uterus- Height [cm]	Mean ± SD	3.31 ± 0.63	3.34 ± 0.65	3.32 ± 0.64	0.346
	Median (quartiles)	3.22 (2.85-3.65)	3.32 (2.9-3.71)	3.26 (2.87-3.68)	
	Range	2.09-5.53	1.75-5.63	1.75-5.63	
Uterus- Width [cm]	Mean ± SD	4.56 ± 0.72	4.56 ± 0.65	4.56 ± 0.7	0.582
	Median (quartiles)	4.53 (4.06-5.05)	4.6 (4.21-4.96)	4.56 (4.12-4.99)	
	Range	2.21-6.89	2.9-6.23	2.21-6.89	
Uterus- Volume [ml]	Mean ± SD	35.49 ± 17.07	36.06 ± 15.8	35.71 ± 16.57	0.303
	Median (quartiles)	31.06 (24.31-42.82)	35.35 (25.18-43.38)	32.15 (24.5-43.01)	
	Range	7.15-118.62	6.04-120.39	6.04-120.39	
Anti-Müllerian hormone [pmol/l]	Mean ± SD	56.03 ± 40.14	45.48 ± 25.93	51.95 ± 35.66	0.004*
	Median (quartiles)	48 (32.32-67.85)	39.2 (28.3-54.7)	44.2 (30.75-62.15)	
	Range	0.16-398	0.07-147	0.07-398	
FSH [mIU/ml]	Mean ± SD	5.91 ± 4.24	5.44 ± 3.96	5.73 ± 4.14	0.022*
	Median (quartiles)	5.75 (4.27-6.91)	5.16 (3.8-6.41)	5.6 (4.12-6.79)	
	Range	1.08-60.6	1.09-44.9	1.08-60.6	
LH [mIU/ml]	Mean ± SD	15.29 ± 10.35	9.61 ± 8.02	13.09 ± 9.9	<0.001*
	Median (quartiles)	13.7 (8.82-18.58)	7.35 (5.28-12.6)	220 (159-410.5)	
	Range	0.3-70.2	0.5-58	0.3-70.2	
Estradiol [pmol/l]	Mean ± SD	360.54 ± 313.38	315.69 ± 342.88	343.2 ± 325.39	0.001*
	Median (quartiles)	231 (174.25-431.25)	186 (123-371)	220 (159-410.5)	
	Range	18-2240	18-2532	18-2532	
Prolactin [μIU/ml]	Mean ± SD	377.99 ± 398.18	320.38 ± 178.45	355.72 ± 331.85	0.026*
	Median (quartiles)	315 (230.75- 420.75)	275 (174-423)	303 (218.5-423)	
	Range	101-5657	49.6-1091	49.6-5657	
Testosterone [nmol/l]	Mean ± SD	2.2 ± 0.59	1.12 ± 0.32	1.78 ± 0.73	<0.001*
	Median (quartiles)	2.11 (1.78- 2.46)	1.17 (0.94-1.38)	1.74 (1.25-2.21)	
	Range	0.93-4.68	0.16-1.66	0.16-4.68	
SHBG [nmol/l]	Mean ± SD	46.55 ± 28.69	58.37 ± 26.83	51.12 ± 28.54	<0.001*
	Median (quartiles)	39.9 (25.9-60.28)	51.3 (41-73.2)	45.8 (30.2-64.5)	
	Range	10.01-200	17.7-200	10.01-200	
FAI [n]	Mean ± SD	6.52 ± 4.29	2.27 ± 1.09	4.88 ± 4	<0.001*
	Median (quartiles)	5.68 (3.41- 8.02)	2.1 (1.41- 3)	3.63 (2.24-6.37)	
	Range	0.88-26.72	0.47-4.98	0.47-26.72	
DHEA-S [μmol/l]	Mean ± SD	10.15 ± 3.78	6.74 ± 2.68	8.83 ± 3.78	<0.001*
	Median (quartiles)	9.24 (7.6-12.48)	6.32 (5.06-8.25)	8.25 (6.23-10.55)	
	Range	2.89-21.2	2.14-20	2.14-21.20	
TSH [μIU/ml]	Mean ± SD	2.13 ± 1.3	2.04 ± 1.07	2.1 ± 1.21	0.361
	Median (quartiles)	1.89 (1.41-2.64)	1.84 (1.35-2.45)	1.87 (1.37-2.59)	
	Range	0.13-14.7	0.16-6.87	0.13-14.7	
fT3 [pmol/l]	Mean ± SD	5.6 ± 0.74	5.2 ± 0.85	5.45 ± 0.81	<0.001*
	Median (quartiles)	5.6 (5.12- 6.12)	5.27 (4.73-5.75)	5.5 (4.94-5.98)	
	Range	2.71-7.6	2.9-7.27	2.71-7.6	
fT4 [pmol/l]	Mean ± SD	16.53 ± 2.5	16.22 ± 2.27	16.41 ± 2.42	0.505
	Median (quartiles)	16.3 (14.9-18.08)	16.1 (14.9-17.8)	16.3 (14.9-17.9)	
	Range	10.4-27.9	8.85-23.9	8.85-27.9	
anti-TPO [IU/ml]	Mean ± SD	26.49 ± 57.3	24 ± 47.93	25.53 ± 53.82	0.264
	Median (quartiles)	12.2 (9-16.25)	12.1 (9-16)	12.1 (9-16.2)	
	Range	9-470	9-411	9-470	
anti-TG [IU/ml]	Mean ± SD	58.95 ± 258.33	43.29 ± 86.45	52.89 ± 209.28	0.906
	Median (quartiles)	16.65 (15.23-18.68)	16.6 (14.9-20.5)	16.6 (15.1-18.9)	
	Range	11.5-3660	11.9-698	11.5-3660	
Fasting glucose [mmol/l]	Mean ± SD	4.89 ± 0.68	4.77 ± 0.54	4.84 ± 0.63	0.053
	Median (quartiles)	4.81 (4.54-5.09)	4.7 (4.47-4.94)	4.77 (4.51-5.04)	
	Range	3.7-11.2	3.66-8.61	3.66-11.2	
120’ OGTT Glucose [mmol/l]	Mean ± SD	5.62 ± 1.52	4.99 ± 1.19	5.37 ± 1.43	<0.001*
	Median (quartiles)	5.45 (4.65-6.43)	4.87 (4.15-5.73)	5.24 (4.4-6.18)	
	Range	2.4-12.2	2.13-9.01	2.13-12.2	
Fasting insulin [μU/ml]	Mean ± SD	13.23 ± 10.79	9.06 ± 5.69	11.62 ± 9.37	<0.001*
	Median (quartiles)	9.74 (6.92-15.28)	7.53 (5.11-10.4)	8.98 (6.36-13.45)	
	Range	2.52-71.9	1.94-31.6	1.94-71.9	
120’ OGTT Insulin [μU/ml]	Mean ± SD	77.39 ± 60.28	51.59 ± 33.77	67.42 ± 53.13	<0.001*
	Median (quartiles)	60 (37.25-99.15)	43.1 (28.3-66.3)	52.2 (33.6-85.95)	
	Range	5.67-387	6.37-229	5.67-387	
HOMA-IR [n]	Mean ± SD	3.03 ± 3.16	1.98 ± 1.45	2.63 ± 2.68	<0.001*
	Median (quartiles)	2.03 (1.45-3.36)	1.6 (1.09- 2.31)	1.89 (1.28-2.95)	
	Range	0.59-24.16	0.37-9.6	0.37-24.16	
Total cholesterol [mmol/l]	Mean ± SD	4.6 ± 0.83	4.66 ± 0.83	4.62 ± 0.83	0.436
	Median (quartiles)	4.6 (4-5)	4.6 (4.1-5.1)	4.6 (4-5.1)	
	Range	2.4-7	2.7-7.6	2.4-7.6	
HDL cholesterol [mmol/l]	Mean ± SD	1.62 ± 0.38	1.76 ± 0.41	1.67 ± 0.4	<0.001*
	Median (quartiles)	1.61 (1.33-1.83)	1.73 (1.46-2.06)	1.64 (1.4-1.89)	
	Range	0.76-2.85	0.91-2.83	0.76-2.85	
LDL cholesterol [mmol/l]	Mean ± SD	2.54 ± 0.7	2.49 ± 0.74	2.52 ± 0.71	0.376
	Median (quartiles)	2.5 (2-3)	2.4 (2-3)	2.5 (2-3)	
	Range	0.7-5	1.1-6.1	0.7-6.1	
Triglycerides [mmol/l]	Mean ± SD	0.98 ± 0.58	0.9 ± 0.42	0.95 ± 0.52	0.349
	Median (quartiles)	0.82 (0.63-1.11)	0.78 (0.6-1.08)	0.81 (0.61-1.1)	
	Range	0.27-4.29	0.34-2.29	0.27-4.29	
AST [IU/l]	Mean ± SD	24.28 ± 9.47	21.91 ± 5.25	23.36 ± 8.18	0.041*
	Median (quartiles)	22 (19-26)	22 (19-25)	22 (19-25)	
	Range	12-94	9-49	9-94	
ALT [IU/l]	Mean ± SD	24.83 ± 15.52	20.14 ± 8.72	23.02 ± 13.49	0.032*
	Median (quartiles)	20 (14-29.75)	18 (15-24)	19 (14.5-26)	
	Range	7-90	6-65	6-90	
CRP [mg/l]	Mean ± SD	2.57 ± 3.41	1.58 ± 1.91	2.19 ± 2.96	0.002*
	Median (quartiles)	1.01 (0.6-3.37)	0.6 (0.6-1.9)	0.83 (0.6-2.55)	
	Range	0.6-20.3	0.6-13.9	0.6-20.3	
Vitamin D [ng/ml]	Mean ± SD	28.97 ± 10.66	31.83 ± 14.22	30.08 ± 12.22	0.167
	Median (quartiles)	28.1 (22- 34.63)	28.3 (23-37.6)	28.2 (22.35-35.7)	
	Range	9.18-76.1	9.95-102	9.18-102	
17-OH Progesterone [ng/ml]	Mean ± SD	1.54 ± 0.93	1.25 ± 0.94	1.43 ± 0.94	<0.001*
	Median (quartiles)	1.27 (0.93-1.87)	0.98 (0.64- 1.5)	1.16 (0.81-1.77)	
	Range	0.28-5.26	0.18-5.97	0.18-5.97	

p, Mann-Whitney test; * statistically significant (p<0.05); SD, standard deviation; quartiles: lower quartile (Q1)- upper quartile (Q3); AMH, anti-Müllerian hormone; FSH, follicle-stimulating hormone; LH, luteinizing hormone; SHBG, sex hormone binding globulin; FAI, free androgen index; DHEA-S, dehydroepiandrosterone sulfate; TSH, thyroid-stimulating hormone; fT3, free triiodothyronine; fT4, free thyroxine; TPOAb, thyroid peroxidase antibodies; TGAb, thyroglobulin antibodies; OGTT, 75-g oral glucose tolerance test; HOMA-IR, homeostasis model assessment for insulin resistance; HDL, high-density lipoprotein; LDL, low-density lipoprotein; AST, aspartate transaminase; ALT, alanine transaminase; CRP, C-reactive protein.

The correlations between biochemical parameters and uterine measurements in women with irregular menstrual cycles were depicted in **Table 4**. The concentrations of AMH, FSH, and AST were negatively correlated with all measurements of the uterine corpus, while LH and HDL cholesterol concentrations were negatively correlated with height, width, and volume, and testosterone concentration was negatively correlated with length, height, and volume measurements (p<0.05, r<0). Estradiol and PRL concentrations were significantly positively correlated with all measurements of the uterine corpus (p<0.05, r>0). The concentrations of AMH, FSH, TC, and HDL cholesterol were significantly negatively correlated with endometrial thickness and volume (p<0.05, r<0), while the concentrations of estradiol, PRL, 17-OHP, FI, and HOMA-IR values were significantly positively correlated with these measures (p<0.05, r>0). Concentrations of other biochemical parameters included in **Table 4** were associated with fewer significant correlations with uterine measurements. There were no significant correlations between the concentrations of SHBG, DHEA-S, fT3, TGAb, FG, LDL cholesterol, TG, CRP, and vitamin D, and any of the assessed uterine ultrasound parameters.

**Table 4:** Correlations between biochemical parameters and uterine measurements in women with irregular menstrual cycles.

Biochemical variable	Endometrium-W [cm]	Endometrium vol. [ml]	Uterus-L [cm]	Uterus-H [cm]	Uterus-W [cm]	Uterus vol. [ml]
Anti-Müllerian hormone [pmol/l]	r=-0.123, p=0.009 *	r=-0.134, p=0.004 *	r=-0.138, p=0.003 *	r=-0.185, p<0.001 *	r=-0.112, p=0.017 *	r=-0.182, p<0.001 *
FSH [mIU/ml]	r=-0.317, p<0.001 *	r=-0.318, p<0.001 *	r=-0.143, p=0.002 *	r=-0.216, p<0.001 *	r=-0.181, p<0.001 *	r=-0.21, p<0.001 *
LH [mIU/ml]	r=-0.013, p=0.777	r=-0.06, p=0.201	r=-0.05, p=0.284	r=-0.147, p=0.002 *	r=-0.131, p=0.005 *	r=-0.135, p=0.004 *
Estradiol [pmol/l]	r=0.564, p<0.001 *	r=0.558, p<0.001 *	r=0.271, p<0.001 *	r=0.297, p<0.001 *	r=0.252, p<0.001 *	r=0.321, p<0.001 *
Prolactin [μIU/ml]	r=0.229, p<0.001 *	r=0.22, p<0.001 *	r=0.121, p=0.01 *	r=0.162, p=0.001 *	r=0.167, p<0.001 *	r=0.174, p<0.001 *
Testosterone [nmol/l]	r=-0.002, p=0.964	r=-0.051, p=0.283	r=-0.134, p=0.004 *	r=-0.146, p=0.002 *	r=-0.058, p=0.22	r=-0.154, p=0.001 *
FAI [n]	r=0.012, p=0.792	r=-0.039, p=0.414	r=-0.123, p=0.009 *	r=-0.082, p=0.081	r=-0.053, p=0.258	r=-0.105, p=0.026 *
TSH [μIU/ml]	r=0.092, p=0.05	r=0.097, p=0.038 *	r=-0.042, p=0.37	r=0.038, p=0.417	r=0.09, p=0.057	r=0.039, p=0.404
fT4 [pmol/l]	r=-0.008, p=0.865	r=0.013, p=0.782	r=0.099, p=0.035 *	r=0.074, p=0.114	r=-0.008, p=0.866	r=0.068, p=0.148
anti-TPO [IU/ml]	r=0.08, p=0.09	r=0.093, p=0.049 *	r=0.022, p=0.637	r=0.068, p=0.147	r=0.01, p=0.83	r=0.036, p=0.444
120’ OGTT Glucose [mmol/l]	r=0.125, p=0.008 *	r=0.075, p=0.112	r=0.005, p=0.922	r=0.071, p=0.131	r=0.018, p=0.698	r=0.036, p=0.439
Fasting insulin [μU/ml]	r=0.151, p=0.001 *	r=0.108, p=0.021 *	r=-0.019, p=0.686	r=0.087, p=0.064	r=0.084, p=0.073	r=0.062, p=0.186
120’ OGTT Insulin [μU/ml]	r=0.113, p=0.016 *	r=0.066, p=0.159	r=-0.012, p=0.804	r=0.059, p=0.214	r=-0.007, p=0.876	r=0.02, p=0.669
HOMA-IR [n]	r=0.141, p=0.003 *	r=0.1, p=0.034 *	r=-0.026, p=0.584	r=0.085, p=0.072	r=0.081, p=0.087	r=0.058, p=0.219
Total cholesterol [mmol/l]	r=-0.126, p=0.007 *	r=-0.169, p<0.001 *	r=-0.071, p=0.13	r=-0.089, p=0.059	r=-0.16, p=0.001 *	r=-0.116, p=0.014 *
HDL cholesterol [mmol/l]	r=-0.246, p<0.001 *	r=-0.231, p<0.001 *	r=-0.07, p=0.139	r=-0.183, p<0.001 *	r=-0.165, p<0.001 *	r=-0.169, p<0.001 *
AST [IU/l]	r=-0.072, p=0.124	r=-0.133, p=0.005 *	r=-0.167, p<0.001 *	r=-0.155, p=0.001 *	r=-0.135, p=0.004 *	r=-0.163, p=0.001 *
ALT [IU/l]	r=-0.044, p=0.347	r=-0.09, p=0.056	r=-0.131, p=0.005 *	r=-0.082, p=0.081	r=-0.073, p=0.121	r=-0.118, p=0.012 *
17-OH Progesterone [ng/ml]	r=0.38, p<0.001 *	r=0.34, p<0.001 *	r=0.081, p=0.084	r=0.145, p=0.002 *	r=0.156, p=0.001 *	r=0.148, p=0.002 *

r, Spearman’s correlation coefficient; * statistically significant (p<0.05); AMH, anti-Müllerian hormone; FSH, follicle-stimulating hormone; LH, luteinizing hormone; FAI, free androgen index; DHEA-S, dehydroepiandrosterone sulfate; TSH, thyroid-stimulating hormone; fT4, free thyroxine; TPOAb, thyroid peroxidase antibodies; OGTT, 75-g oral glucose tolerance test; HOMA-IR, homeostasis model assessment for insulin resistance; HDL, high-density lipoprotein; AST, aspartate transaminase; ALT, alanine transaminase.

The correlations between selected clinical quantitative variables and uterine dimensions in women with irregular and regular menstrual cycles were delineated in **Supplementary Table 1**. In women with regular menstrual cycles, all uterine measurements positively correlated with age (p=0.41 for endometrial thickness and p=0.007 for volume; p<0.001 for uterine length, height, width, and volume). Conversely, in women with irregular cycles, endometrial thickness and volume showed no correlations with age (all p>0.05), whereas other measurements exhibited analogous relationships to those in women with regular cycles. Endometrial thickness positively correlated with BMI only in women with irregular menstrual cycles (p=0.003). Uterine height (p<0.001), width (p=0.034), and volume (p=0.006) showed positive correlations with BMI in women with irregular cycles, as did uterine height (p<0.001) and volume (p=0.016) in women with regular menstrual cycles. In women with irregular menstrual cycles, the average cycle length negatively correlated with all uterine measurements (all p<0.001), whereas in those with regular menstruation no correlation was found between uterine dimensions and cycle length (all p>0.05). Ovarian volume exhibited positive correlations solely with endometrial thickness and volume, which were especially pronounced in women with irregular menstruation. In women with irregular cycles, the volumes of the right (p<0.001) and left (p=0.015) ovaries positively correlated with endometrial thickness.

The correlations between selected clinical qualitative variables and uterine dimensions in women with irregular and regular menstrual cycles were delineated in **Supplementary Table 2**. In both study arms, uterine dimensions increased with the number of pregnancies (most p<0.001). Regarding endometrial measurements, the number of pregnancies correlated only with endometrial volume in women with regular cycles (p=0.002). Similar correlations in both groups were observed for the number of childbirths concerning the same uterine parameters. No association was found between dysmenorrhea and uterine measurements in women with irregular cycles (all p>0.05). However, among women with regular cycles, those with dysmenorrhea had smaller uterine length (p=0.03) and height (p=0.012). In women with irregular cycles, those with HMB had significantly thicker endometrium compared to those without AUB (p=0.024). Other uterine measurements did not correlate with AUB in either group (all p>0.05). In women with irregular cycles, all uterine measurements were significantly reduced in those identified with PCOM (all p<0.05), whereas in women with regular cycles, only uterine height and width were significantly smaller (p=0.024 and p=0.003). In women with irregular cycles presenting with a dominant follicle, endometrial thickness and volume (both p<0.001), as well as uterine height (p=0.007), width (p=0.011), and volume (p=0.005), were significantly greater than in those without a dominant follicle. In contrast, no such relationships were found among women with regular cycles (all p>0.05).

## 4 Discussion

Circulating biochemical substances that reflect the body’s hormonal and metabolic status regulate the myometrium and endometrium. These substances are key drivers of cyclical processes such as endometrial growth, differentiation, receptivity, and myometrial contractility, while also impacting pathologies such as endometrial hyperplasia, cancer, adenomyosis, and fibroids through intricate endocrine and metabolic signaling pathways (7, 12). The study found that women with irregular menstrual cycles exhibited significantly reduced uterine and endometrial dimensions, along with increased ovarian volume and a higher prevalence of PCOM, compared to those with regular cycles. Nevertheless, the literature on this topic is scarce and largely confined to comparisons between women with PCOS and healthy controls (13–16).

### 4.1 Myometrial and endometrial measurements in women with normogonadotropic anovulation and healthy women

As demonstrated in this study, hormonal and metabolic disturbances – manifested by hyperandrogenism, increased BMI, prolonged menstrual cycles, and PCOM – affected the myometrium and endometrium in women with ovulatory disorders, resulting in a decrease in all uterine measurements compared to healthy women. The findings of reduced endometrial thickness in women with PCOS in this study aligned with prior limited research on the endometrium in women with PCOS and oligomenorrhea, attributing this effect to the inhibitory influence of androgens (15). However, it should be noted that a number of earlier studies indicated that the endometrium was thicker in women with PCOS compared to healthy controls, which was attributed to unbalanced estrogen stimulation resulting from an ovulation defect (17–19), while a large prospective study did not show significant differences (14). It is widely acknowledged that impaired endometrial receptivity in PCOS, likely arising from hyperandrogenism, inflammation, insulin resistance, and obesity, along with ovulatory dysfunction, constitutes a principal factor contributing to infertility in this condition (20). Although the correlation between endometrial receptivity and thickness has not been directly studied in PCOS (21, 22), it has been demonstrated that decreased endometrial thickness in PCOS was significantly associated with obstetric complications such as preterm birth, low birth weight, and small-for-gestational age (23). Addressing the issues of endometrial dysfunction and reduced thickness in PCOS may be facilitated by research involving endometrial epithelial organoids, which have demonstrated decreased volume in PCOS compared to healthy controls, as well as increased expression of inflammation-related genes and abnormal responses to ovarian steroid hormones (24). Data regarding the myometrium in women with PCOS, in contrast, are more limited, with one study not showing significant changes compared to healthy controls (15), while another reported a reduction in myometrial thickness in women with PCOS relative to healthy individuals (16).

The comparative analysis of uterine and endometrial morphology between women with HPOD and healthy women has not received adequate research attention, resulting in a lack of published data for comparative evaluation of the findings.

In women with normogonadotropic anovulation, the thickness of the endometrium significantly increased with rising BMI, increased ovarian volume, and the presence of HMB, whereas it negatively correlated with the length of the menstrual cycle and the presence of PCOM. In contrast, in regularly menstruating women, endometrial thickness positively correlated with age and increased ovarian volume likely resulting from a dominant follicle, while its volume positively correlated with the number of pregnancies and deliveries.

Intergroup differences in correlations related to the myometrium were less pronounced than those for the endometrium. In both groups, nearly all myometrial measurements correlated significantly with age, BMI, and the number of pregnancies and deliveries. The observations of the increase in uterine dimensions with age were consistent with previous findings in both women with PCOS and healthy women (14). In women with normogonadotropic anovulation, a negative correlation was observed with cycle length and a positive correlation with the presence of a physiological ovarian structure. However, no significant correlations were found between myometrial measurements and ovarian volume in either group.

While metabolic and hormonal processes, along with the ultrasonographic characteristics of the endometrium, have been extensively investigated – specifically in the context of PCOS – due to their direct associations with abnormal uterine bleeding, neoplasia, infertility, and obstetric complications (7), knowledge regarding the myometrium in the context of normogonadotropic anovulation remains comparatively limited.

### 4.2 Myometrial and endometrial measurements in women with polycystic ovary syndrome and hypothalamic-pituitary-ovarian axis dysfunction

Among women with normogonadotropic anovulation, those diagnosed with PCOS exhibited a further reduction in uterine dimensions compared to women with HPOD, while endometrial measurements remained comparable between the two conditions, with this finding likely associated with variations in the assessed concentrations of biochemical factors. Notably, the concentrations of AMH, LH and testosterone, which showed negative correlations with myometrial measurements, were higher in women with PCOS, while the concentration of estradiol, which positively correlated with uterine dimensions, was lower in this population. Particularly intriguing in this context was the unexpectedly significantly lower concentration of estradiol in women with PCOS compared to those with HPOD, for which previous studies established higher concentrations in PCOS compared to healthy women (25), although there were also opposing reports (7).

Data on the influence of PCOS on the morphology and function of the myometrium are limited (13). Although the literature has reported alterations in uterine peristalsis in women with PCOS, some studies have indicated no differences in myometrial morphology between women with PCOS and the control group (14, 15), while other research has shown a reduction in myometrial thickness in women with PCOS compared to healthy controls (16). In a study examining ultrasonographic characteristics of endometrial thickness and uterine measurements in adolescent girls with PCOS, it was found that endometrial thickness was increased - likely due to unopposed estrogen exposure - while uterine length was shorter, which the authors hypothesized could result from a chronic imbalance between estrogen and androgens (26).

Interestingly, no significant differences were found in endometrial measurements between women with PCOS and those with HPOD in this study, although the concentration of AMH, which negatively correlated with endometrial thickness, was higher in women with PCOS, while the concentration of estradiol, which positively correlated with endometrial thickness, was lower. Therefore, it appears that the proliferative effects of metabolic factors such as insulin resistance, hyperinsulinemia, and elevated concentration of 17-OHP have overshadowed the impact of hormonal differences related to estradiol and AMH in women with PCOS.

The comparative analysis of myometrial and endometrial dimensions and volumes between women with PCOS and HPOD is unique to this study. The literature lacks comparative data on myometrial measurements in women with PCOS and HPOD, as these parameters have not been explored in this context. Consequently, it is not feasible to interpret the results in the context of previous research.

### 4.3 Myometrial and endometrial measurements in polycystic ovary syndrome with and without hyperandrogenemia

Hyperandrogenemia in PCOS was specifically associated with increased endometrial thickness, underscoring the modulatory effect of androgen excess on the endometrium, while not proportionally influencing overall uterine size. Previous scientific evidence has demonstrated that androgens exert direct antiproliferative effects on endometrial epithelial and stromal cells, likely by antagonizing the action of estrogens (27, 28). Endometrial cells expressing the androgen receptor (AR) serve as target sites for circulating and locally produced androgens, which are essential for their cyclical function (29). AR synthesis is spatially and temporally regulated, predominantly expressed in stromal cells, peaking during the proliferative phase, reflecting the promoting role of estrogens and the inhibitory effect of progesterone on AR expression. Conversely, AR expression in epithelial cells significantly increases during the late secretory phase, coinciding with a decline in circulating progesterone concentration (27). Research has demonstrated that long-term androgen administration is associated with increased AR expression in the endometrial stroma and myometrium, suggesting an anticipatory mechanism of androgen action. In women with PCOS, AR protein production is upregulated in endometrial glands and stroma during both the proliferative and secretory phases (27). However, in certain studies no differences were found in endometrial thickness between women with PCOS and healthy controls, nor between women with PCOS and hyperandrogenemia and those with PCOS without hyperandrogenemia (14, 15). Interestingly, this study did not confirm a significant negative correlation between testosterone concentration and endometrial thickness in women with normogonadotropic anovulation. Indeed, when comparing women with PCOS, characterized by elevated testosterone concentration, to women with HPOD, no significant differences in endometrial thickness and volume were observed. Moreover, in women with PCOS, those with hyperandrogenemia had surprisingly thicker endometrium compared to those with normoandrogenemia. Conversely, AMH concentration, previously demonstrated to positively correlate with testosterone in PCOS (30), correlated negatively with endometrial thickness in women with normogonadotropic anovulation. The results of this study indicated that the significantly elevated concentrations of substances such as estradiol, FI, and PRL in cases of hyperandrogenemia had a substantial impact on endometrial thickening in this subset of women, likely outweighing the negative influence of increased concentrations of AMH and FSH on endometrial thickness.

In contrast to the endometrial characteristics, no significant differences were detected in myometrial measurements between women with PCOS and hyperandrogenemia and those with PCOS who had normal testosterone concentrations. However, it was previously demonstrated in one study that women with hyperandrogenemia in PCOS had reduced uterine dimensions compared to their counterparts without hyperandrogenemia (14).

### 4.4 Myometrial and endometrial measurements and metabolic factors

The literature has reported that metabolic imbalances in carbohydrate metabolism, and to a lesser extent in lipid metabolism (31, 32), affect uterine morphology and function through disturbances in steroid hormone signaling, disrupted insulin and leptin pathways, chronic low-grade inflammation, and oxidative stress (25). It has been previously demonstrated (33) that hyperinsulinemia and hyperandrogenism – through correlations between the concentrations of FI, 120’OGTTI, and FAI values with C-reactive protein (CRP) and interleukin-6 (IL-6) – intensify low-grade systemic inflammation, yet no significant correlations were found between these inflammatory markers and endometrial thickness. Additionally, no significant correlations were found between the concentrations of CRP, IL-6, and testosterone, contradicting claims from other studies (31). It can therefore be inferred that metabolic factors may affect the endometrium through a mechanism that is independent of pro-inflammatory activity and testosterone. Prior research has revealed that resistance-induced hyperinsulinemia may promote endometrial proliferation by interfering with the cell cycle (34) and through signaling pathways associated with insulin-like growth factors, vascular endothelial growth factor, estrogens, and their respective receptors (35). Observations of the direct interaction of insulin-dependent signaling pathways on the proliferation of endometrial cells were reflected in the study’s results, which showed a significant correlation between FI concentrations and HOMA-IR values and endometrial thickness, without any correlations found for testosterone, LH, or SHBG concentrations. One of the preceding investigations did not establish a correlation between LH concentration or BMI values and endometrial thickness in women with PCOS (36).

### 4.5 Conclusions

The results of the study have demonstrated a substantial association between menstrual cycle regularity and uterine morphology. Correlation analyses revealed that myometrial and endometrial measurements in women with normogonadotropic anovulation were significantly influenced by hormonal, metabolic, and clinical factors. Negative correlations between myometrial measurements and concentrations of AMH, gonadotropins, and testosterone, coupled with positive correlations with estradiol and prolactin concentrations, underscored the pivotal role of estrogenic stimulation in uterine growth and maintenance. Positive correlations between endometrial measurements and markers of insulin resistance emphasized the important influence of metabolic status on endometrial morphology. Clinical factors, including age, BMI, reproductive history, menstrual cycle length, ovarian volume, and the presence of a dominant follicle or PCOM, further modulated uterine and endometrial dimensions, with more pronounced and complex relationships in women with normogonadotropic anovulation. Collectively, these findings have indicated that uterine morphology in women with ovulatory disorders reflects the cumulative effects of reproductive history and chronic endocrine-metabolic alterations rather than isolated hormonal abnormalities. Overall, this study provides comprehensive evidence that menstrual irregularity and PCOS are associated with distinct patterns of uterine and endometrial morphology. These findings have potential clinical implications for reproductive counseling, fertility assessment, and long-term gynecological follow-up in women with PCOS and HPOD, highlighting the value of uterine morphology as an additional marker of chronic endocrine dysfunction. Advancements in imaging techniques, coupled with a deeper understanding of the relationships between imaging and biochemical data, are crucial for effectively managing gynecological disorders and improving reproductive health outcomes.

### 4.6 Directions for future research

Future research should prioritize increasing participant numbers across all research arms, expanding the study to encompass both phases of the menstrual cycle, and investigating the molecular mechanisms underlying the observed phenomena. Given the limited understanding of the biochemical processes regulating the endometrium and myometrium, it is challenging to accurately infer their functional states based solely on ultrasound evaluations. Insights into the complex interactions influenced by steroid hormones and insulin in women’s reproductive health primarily stem from studies on PCOS, androgen hormone replacement therapy in transgender men, reduced ovarian reserve, and animal models (28, 37). Therefore, further studies are essential to clarify the roles of these factors in the physiology of the endometrium and myometrium (38), as this understanding will enhance knowledge of the interactions between these variables and facilitate the development of targeted therapies.

### 4.7 Strengths and weaknesses of the study

The study exhibits several strengths, including a robust methodology, a multifaceted approach that integrates both quantitative and qualitative analyses of carefully selected variables, and an adequate sample size, which enhances the generalizability of the findings and their potential clinical implications, thereby contributing to improvements in medical practice. Nonetheless, it is important to acknowledge certain limitations, such as temporal constraints, a single-center design, and unequal sample sizes across research arms, which may impact the representativeness of the results. Additionally, focusing on non-malformed uteri, the study did not assess the incidence of fibroids or congenital uterine malformations (39), as these were established as exclusion criteria.

## Data Availability

The datasets presented in this study can be found in online repositories. The names of the repository/repositories and accession number(s) can be found below: https://doi.org/10.7910/DVN/HZNRLK.
