# Supplementary figures and images for: Uterine morphology in normogonadotropic anovulation: a comparative study of polycystic ovary syndrome and hypothalamic-pituitary-ovarian dysfunction
Source: Front Endocrinol (Lausanne). 2026 Apr 2;17:1781593. doi: 10.3389/fendo.2026.1781593 (PMC13082961; doi:10.3389/fendo.2026.1781593)

1.1.

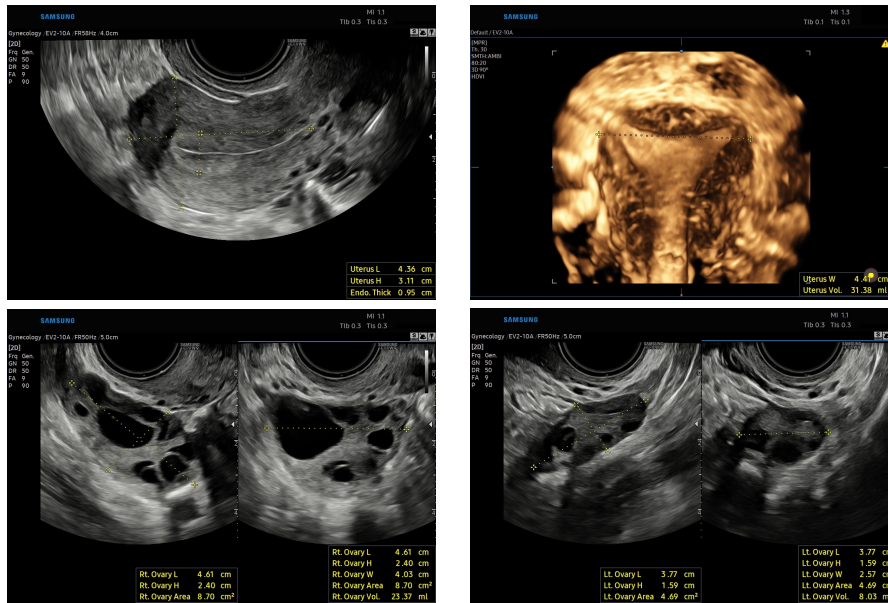

1.2.

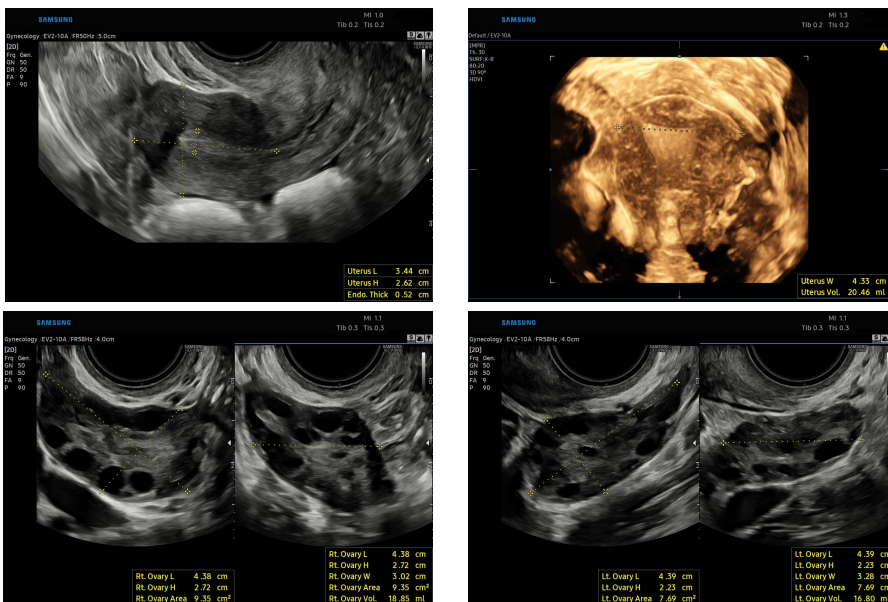

1.3.

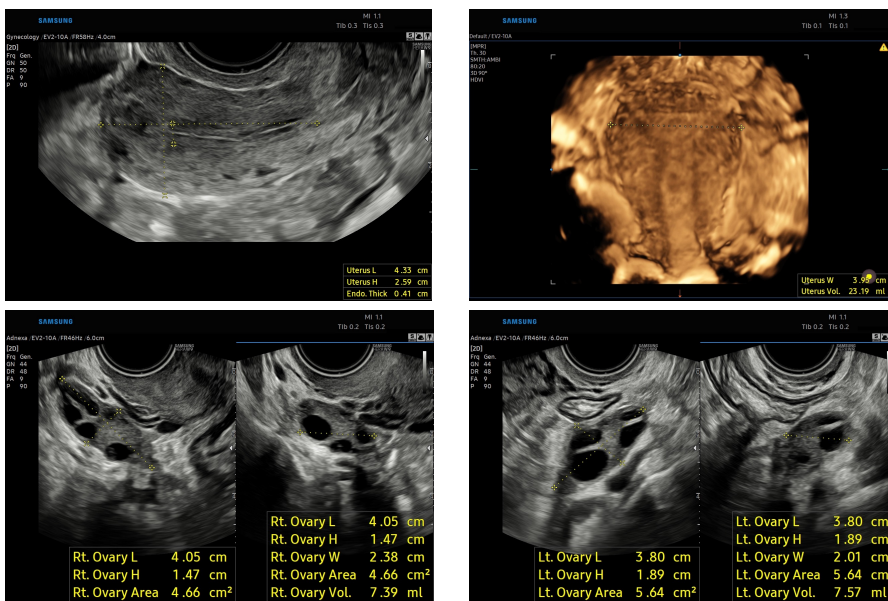

Supplement: Supplementary file 1 [file DataSheet1.pdf]
