# Supplementary material for: Uterine morphology in normogonadotropic anovulation: a comparative study of polycystic ovary syndrome and hypothalamic-pituitary-ovarian dysfunction
Source: Front Endocrinol (Lausanne). 2026 Apr 2;17:1781593. doi: 10.3389/fendo.2026.1781593 (PMC13082961; doi:10.3389/fendo.2026.1781593)
Supplement: Supplementary file 2 [file Table1.docx]

Supplementary Material

# Supplementary Tables

Supplementary Table 1. Correlations between selected clinical quantitative variables and uterine dimensions in women with irregular menstrual cycles, regular cycles, and within the entire study cohort.

| **Parameter** | **Study group** | **Endometrium-Thickness [cm]** | **Endometrium- Volume [ml]** | **Uterus- Length [cm]** | **Uterus- Height[cm]** | **Uterus- Width [cm]** | **Uterus- Volume [ml]** |
| --- | --- | --- | --- | --- | --- | --- | --- |
| Age [years] | Irregular cycles | r=0.054,  p=0.254 | r=0.08, p=0.088 | r=0.224, p<0.001* | r=0.256, p<0.001* | r=0.124, p=0.009* | r=0.256, p<0.001* |
|  | Regular cycles | r=0.146, p=0.041* | r=0.191, p=0.007* | r=0.3, p<0.001* | r=0.367, p<0.001* | r=0.291, p<0.001* | r=0.36, p<0.001* |
|  | Total cohort | r=0.118, p=0.003* | r=0.141, p<0.001* | r=0.257, p<0.001* | r=0.311, p<0.001* | r=0.193, p<0.001* | r=0.304, p<0.001* |
| BMI [kg/m²] | Irregular cycles | r=0.137, p=0.003* | r=0.079, p=0.092 | r=0.056, p=0.231 | r=0.171, p<0.001* | r=0.1,  p=0.034* | r=0.13, p=0.006* |
|  | Regular cycles | r=0.113,  p=0.115 | r=0.085, p=0.235 | r=0.072, p=0.315 | r=0.264, p<0.001* | r=0.101, p=0.158 | r=0.172, p=0.016* |
|  | Total cohort | r=0.098, p=0.013* | r=0.05, p=0.207 | r=0.041, p=0.293 | r=0.165, p<0.001* | r=0.08,  p=0.042* | r=0.113, p=0.004* |
| Average cycle  length [days] | Irregular cycles | r=-0.175, p<0.001* | r=-0.208, p<0.001* | r=-0.221, p<0.001* | r=-0.199, p<0.001* | r=-0.157, p=0.001* | r=-0.231, p<0.001* |
|  | Regular cycles | r=0.008,  p=0.916 | r=0,  p=0.997 | r=-0.019, p=0.793 | r=-0.03, p=0.673 | r=-0.029, p=0.685 | r=-0.039, p=0.588 |
|  | Total cohort | r=-0.21,  p<0.001* | r=-0.251, p<0.001* | r=-0.221, p<0.001* | r=-0.237, p<0.001* | r=-0.199, p<0.001* | r=-0.258, p<0.001* |
| Right Ovarian  Volume [ml] | Irregular cycles | r=0.172, p<0.001* | r=0.18, p<0.001* | r=0.089, p=0.06 | r=0.089, p=0.059 | r=0.063,  p=0.18 | r=0.08, p=0.091 |
|  | Regular cycles | r=0.08,  p=0.268 | r=0.134, p=0.061 | r=-0.029, p=0.691 | r=0.025, p=0.729 | r=0.032, p=0.656 | r=0.018, p=0.799 |
|  | Total cohort | r=0.127, p=0.001* | r=0.146, p<0.001* | r=0.048, p=0.226 | r=0.053, p=0.176 | r=0.051, p=0.195 | r=0.051, p=0.191 |
| Left Ovarian  Volume [ml] | Irregular cycles | r=0.114, p=0.015* | r=0.075, p=0.113 | r=-0.016, p=0.739 | r=0.041, p=0.38 | r=0.075, p=0.111 | r=0.04, p=0.397 |
|  | Regular cycles | r=0.159, p=0.026* | r=0.193, p=0.007* | r=-0.061, p=0.396 | r=-0.074, p=0.305 | r=-0.053, p=0.463 | r=-0.061, p=0.393 |
|  | Total cohort | r=0.102, p=0.009* | r=0.085, p=0.031* | r=-0.041, p=0.303 | r=-0.016, p=0.677 | r=0.025, p=0.522 | r=-0.009, p=0.826 |

r= Spearman's correlation coefficient, * statistically significant (p<0.05), BMI= body mass index

Supplementary Table 2 [*designed for horizontal arrangement*]. Correlations between selected clinical qualitative variables and uterine dimensions in women with irregular menstrual cycles, regular cycles, and within the entire study cohort.

| **Parameter** | | **Value** | **Irregular menstrual cycles** | | | | | | | | | **Regular menstrual cycles** | | | | | | | | | **Total cohort** | | | | | | | | |
| --- | --- | --- | --- | --- | --- | --- | --- | --- | --- | --- | --- | --- | --- | --- | --- | --- | --- | --- | --- | --- | --- | --- | --- | --- | --- | --- | --- | --- | --- |
|  |  |  | N | Mean | SD | Median | Min | Max | Q1 | Q3 | p | N | Mean | SD | Median | Min | Max | Q1 | Q3 | p | N | Mean | SD | Median | Min | Max | Q1 | Q3 | p |
| Pregnancies^a^ | Endometrium- Thickness [cm] | 0 (A) | 401 | 0.73 | 0.34 | 0.68 | 0.1 | 2.1 | 0.48 | 0.93 | p=0.885 | 157 | 0.85 | 0.33 | 0.87 | 0.15 | 1.8 | 0.62 | 1.04 | p=0.054 | 558 | 0.76 | 0.34 | 0.73 | 0.1 | 2.1 | 0.51 | 0.99 | p=0.162 |
|  |  | 1 (B) | 20 | 0.78 | 0.35 | 0.72 | 0.3 | 1.49 | 0.51 | 1.06 |  | 19 | 0.93 | 0.28 | 0.98 | 0.52 | 1.42 | 0.68 | 1.14 |  | 39 | 0.86 | 0.32 | 0.85 | 0.3 | 1.49 | 0.56 | 1.14 |  |
|  |  | 2 (C) | 20 | 0.7 | 0.25 | 0.65 | 0.43 | 1.24 | 0.47 | 0.81 |  | 13 | 1 | 0.33 | 0.98 | 0.5 | 1.68 | 0.86 | 1.08 |  | 33 | 0.82 | 0.32 | 0.77 | 0.43 | 1.68 | 0.56 | 1.03 |  |
|  |  | ≥ 3 (D) | 11 | 0.7 | 0.32 | 0.51 | 0.33 | 1.18 | 0.44 | 0.97 |  | 7 | 0.65 | 0.17 | 0.71 | 0.44 | 0.87 | 0.5 | 0.77 |  | 18 | 0.68 | 0.26 | 0.62 | 0.33 | 1.18 | 0.47 | 0.85 |  |
|  | Endometrium- volume [ml] | 0 (A) | 401 | 2.95 | 2.34 | 2.23 | 0.12 | 16.28 | 1.35 | 3.85 | p=0.663 | 157 | 3.69 | 2.3 | 3.34 | 0.22 | 13.39 | 2.05 | 4.82 | p=0.006* | 558 | 3.15 | 2.35 | 2.49 | 0.12 | 16.28 | 1.49 | 4.21 | p=0.009 * |
|  |  | 1 (B) | 20 | 2.97 | 1.8 | 2.42 | 0.26 | 7.46 | 1.64 | 4.43 |  | 19 | 5.12 | 2.55 | 5.23 | 1.88 | 9.91 | 2.64 | 7.18 | B>A,D C,A>D | 39 | 4.01 | 2.43 | 3.23 | 0.26 | 9.91 | 2.16 | 5.28 | C>D B>A,D |
|  |  | 2 (C) | 20 | 3.07 | 1.75 | 2.64 | 1.07 | 7.93 | 1.71 | 4.05 |  | 13 | 4.34 | 1.69 | 3.76 | 1.72 | 7.75 | 3.4 | 4.58 |  | 33 | 3.57 | 1.82 | 3.4 | 1.07 | 7.93 | 2.04 | 4.3 |  |
|  |  | ≥ 3 (D) | 11 | 3.15 | 3.25 | 1.73 | 0.82 | 10.91 | 1.17 | 3.05 |  | 7 | 2.18 | 0.87 | 1.68 | 1.53 | 3.6 | 1.58 | 2.63 |  | 18 | 2.77 | 2.59 | 1.71 | 0.82 | 10.91 | 1.54 | 3.14 |  |
|  | Uterus- Length [cm] | 0 (A) | 401 | 4.26 | 0.71 | 4.22 | 1.87 | 6.55 | 3.81 | 4.7 | p=0.002* | 157 | 4.4 | 0.6 | 4.37 | 2.69 | 6.19 | 4.03 | 4.79 | p<0.001* | 558 | 4.3 | 0.68 | 4.27 | 1.87 | 6.55 | 3.85 | 4.72 | p<0.001 * |
|  |  | 1 (B) | 20 | 4.47 | 0.71 | 4.34 | 3.3 | 6.07 | 3.97 | 4.97 | C>B,A | 19 | 5.05 | 0.7 | 5.2 | 3.75 | 6.16 | 4.57 | 5.3 | B>A,D C>A | 39 | 4.75 | 0.76 | 4.61 | 3.3 | 6.16 | 4.11 | 5.28 | C>A,D B>A |
|  |  | 2 (C) | 20 | 4.96 | 0.72 | 4.88 | 3.89 | 6.15 | 4.43 | 5.55 |  | 13 | 4.92 | 0.69 | 5.07 | 3.61 | 5.82 | 4.38 | 5.47 |  | 33 | 4.94 | 0.7 | 4.94 | 3.61 | 6.15 | 4.38 | 5.54 |  |
|  |  | ≥ 3 (D) | 11 | 4.63 | 1 | 4.21 | 3.64 | 6.94 | 4.14 | 4.72 |  | 7 | 4.41 | 0.38 | 4.21 | 4.06 | 5 | 4.12 | 4.68 |  | 18 | 4.54 | 0.81 | 4.21 | 3.64 | 6.94 | 4.12 | 4.78 |  |
|  | Uterus- Height [cm] | 0 (A) | 401 | 3.3 | 0.63 | 3.24 | 1.75 | 5.63 | 2.86 | 3.66 | p<0.001* | 157 | 3.52 | 0.61 | 3.48 | 1.07 | 5.76 | 3.12 | 3.84 | p<0.001* | 558 | 3.36 | 0.63 | 3.3 | 1.07 | 5.76 | 2.91 | 3.71 | p<0.001 * |
|  |  | 1 (B) | 20 | 3.76 | 0.78 | 3.58 | 2.48 | 5.53 | 3.44 | 4.32 | C,D,B>A | 19 | 3.99 | 0.5 | 3.94 | 3.15 | 5.1 | 3.73 | 4.27 | C,B>A | 39 | 3.87 | 0.66 | 3.82 | 2.48 | 5.53 | 3.5 | 4.3 | C,B,D>A |
|  |  | 2 (C) | 20 | 3.95 | 0.59 | 4.14 | 2.44 | 4.55 | 3.66 | 4.39 |  | 13 | 4.15 | 0.73 | 4.33 | 2.72 | 4.98 | 3.73 | 4.8 |  | 33 | 4.03 | 0.65 | 4.19 | 2.44 | 4.98 | 3.72 | 4.47 |  |
|  |  | ≥ 3 (D) | 11 | 3.9 | 0.78 | 3.63 | 2.67 | 5.6 | 3.49 | 4.29 |  | 7 | 3.67 | 0.28 | 3.75 | 3.32 | 4.03 | 3.44 | 3.87 |  | 18 | 3.81 | 0.63 | 3.69 | 2.67 | 5.6 | 3.47 | 4.01 |  |
|  | Uterus- Width [cm] | 0 (A) | 401 | 4.52 | 0.68 | 4.52 | 2.21 | 6.89 | 4.08 | 4.9 | p<0.001* | 157 | 4.72 | 0.68 | 4.63 | 2.71 | 6.93 | 4.27 | 5.1 | p<0.001* | 558 | 4.57 | 0.68 | 4.56 | 2.21 | 6.93 | 4.13 | 5.01 | p<0.001 * |
|  |  | 1 (B) | 20 | 4.97 | 0.72 | 4.86 | 3.72 | 6.75 | 4.5 | 5.46 | D,B,C>A | 19 | 5.31 | 0.66 | 5.28 | 4.33 | 6.48 | 4.86 | 5.78 | C,B>A | 39 | 5.14 | 0.71 | 4.96 | 3.72 | 6.75 | 4.58 | 5.69 | D,B,C>A |
|  |  | 2 (C) | 20 | 4.84 | 0.84 | 4.8 | 1.95 | 6.2 | 4.59 | 5.37 |  | 13 | 5.24 | 0.6 | 5.41 | 3.97 | 6.05 | 4.81 | 5.6 |  | 33 | 5 | 0.77 | 4.9 | 1.95 | 6.2 | 4.68 | 5.44 |  |
|  |  | ≥ 3 (D) | 11 | 4.94 | 0.77 | 5.05 | 3.05 | 5.9 | 4.7 | 5.39 |  | 7 | 4.91 | 0.5 | 4.85 | 4.29 | 5.61 | 4.6 | 5.22 |  | 18 | 4.93 | 0.66 | 4.99 | 3.05 | 5.9 | 4.67 | 5.43 |  |
|  | Uterus- volume [ml] | 0 (A) | 401 | 34.85 | 15.58 | 31.88 | 6.04 | 120.39 | 24.25 | 42.31 | p<0.001* | 157 | 39.52 | 15.48 | 37.48 | 5.44 | 117.82 | 29.31 | 46.01 | p<0.001* | 558 | 36.16 | 15.68 | 33.26 | 5.44 | 120.39 | 25.6 | 43.31 | p<0.001 * |
|  |  | 1 (B) | 20 | 46.86 | 24.47 | 38.59 | 19.97 | 118.62 | 34.26 | 53.68 | C,D,B>A | 19 | 56.26 | 15.18 | 58.9 | 25.87 | 76.02 | 49.88 | 65.32 | C,B>A | 39 | 51.44 | 20.77 | 49.45 | 19.97 | 118.62 | 37.19 | 65.24 | C,B,D>A |
|  |  | 2 (C) | 20 | 53.29 | 15.62 | 50.92 | 28.37 | 76.67 | 39.88 | 67.46 |  | 13 | 58.4 | 20.68 | 62.84 | 23.98 | 89.67 | 40.8 | 73.42 |  | 33 | 55.3 | 17.66 | 56.94 | 23.98 | 89.67 | 40.46 | 69.71 |  |
|  |  | ≥ 3 (D) | 11 | 49.81 | 25.36 | 46.03 | 15.53 | 100.61 | 36.44 | 50.66 |  | 7 | 41.69 | 7.18 | 39.74 | 33.58 | 54.83 | 37.64 | 44.21 |  | 18 | 46.65 | 20.33 | 40.37 | 15.53 | 100.61 | 37.23 | 49.13 |  |
| Childbirths^a^ | Endometrium- Thickness [cm] | 0 (A) | 411 | 0.73 | 0.34 | 0.68 | 0.1 | 2.1 | 0.48 | 0.92 | p=0.49 | 174 | 0.86 | 0.33 | 0.87 | 0.15 | 1.8 | 0.62 | 1.04 | p=0.242 | 585 | 0.76 | 0.34 | 0.73 | 0.1 | 2.1 | 0.51 | 0.99 | p=0.114 |
|  |  | 1 (B) | 19 | 0.82 | 0.35 | 0.75 | 0.3 | 1.49 | 0.53 | 1.12 |  | 12 | 0.97 | 0.25 | 1.02 | 0.52 | 1.33 | 0.85 | 1.13 |  | 31 | 0.87 | 0.32 | 0.88 | 0.3 | 1.49 | 0.6 | 1.14 |  |
|  |  | ≥2 (C) | 22 | 0.7 | 0.27 | 0.65 | 0.42 | 1.24 | 0.46 | 0.88 |  | 10 | 0.82 | 0.38 | 0.77 | 0.44 | 1.68 | 0.53 | 0.96 |  | 32 | 0.74 | 0.31 | 0.66 | 0.42 | 1.68 | 0.48 | 0.91 |  |
|  | Endometrium- volume [ml] | 0 (A) | 411 | 2.93 | 2.32 | 2.21 | 0.12 | 16.28 | 1.35 | 3.83 | p=0.417 | 174 | 3.73 | 2.28 | 3.37 | 0.22 | 13.39 | 2.07 | 4.78 | p=0.037* | 585 | 3.16 | 2.33 | 2.5 | 0.12 | 16.28 | 1.56 | 4.24 | p=0.068 |
|  |  | 1 (B) | 19 | 3.11 | 1.84 | 2.45 | 0.26 | 7.46 | 1.88 | 4.44 |  | 12 | 5.48 | 2.48 | 5.36 | 1.88 | 8.79 | 3.79 | 7.47 | B>A,C | 31 | 4.03 | 2.38 | 4.04 | 0.26 | 8.79 | 2.2 | 5.28 |  |
|  |  | ≥2 (C) | 22 | 3.41 | 2.59 | 2.73 | 1.07 | 10.91 | 1.59 | 4.04 |  | 10 | 3.35 | 2.03 | 3.33 | 1.53 | 7.75 | 1.61 | 3.66 |  | 32 | 3.4 | 2.4 | 2.88 | 1.07 | 10.91 | 1.59 | 3.94 |  |
|  | Uterus- Length [cm] | 0 (A) | 411 | 4.26 | 0.7 | 4.2 | 1.87 | 6.55 | 3.8 | 4.7 | p<0.001 * | 174 | 4.44 | 0.6 | 4.38 | 2.69 | 6.19 | 4.08 | 4.88 | p=0.008* | 585 | 4.31 | 0.68 | 4.28 | 1.87 | 6.55 | 3.87 | 4.73 | p<0.001 * |
|  |  | 1 (B) | 19 | 4.74 | 0.81 | 4.64 | 3.3 | 6.15 | 4 | 5.34 | C,B>A | 12 | 5.08 | 0.84 | 5.2 | 3.75 | 6.16 | 4.53 | 5.85 | B>A | 31 | 4.87 | 0.83 | 4.96 | 3.3 | 6.16 | 4.14 | 5.44 | B,C>A |
|  |  | ≥2 (C) | 22 | 4.97 | 0.79 | 4.84 | 3.92 | 6.94 | 4.46 | 5.57 |  | 10 | 4.88 | 0.72 | 4.96 | 4.06 | 5.82 | 4.18 | 5.51 |  | 32 | 4.94 | 0.76 | 4.85 | 3.92 | 6.94 | 4.31 | 5.6 |  |
|  | Uterus- Height [cm] | 0 (A) | 411 | 3.3 | 0.62 | 3.24 | 1.75 | 5.63 | 2.87 | 3.66 | p<0.001* | 174 | 3.55 | 0.62 | 3.51 | 1.07 | 5.76 | 3.14 | 3.87 | p<0.001* | 585 | 3.38 | 0.63 | 3.34 | 1.07 | 5.76 | 2.92 | 3.72 | p<0.001 * |
|  |  | 1 (B) | 19 | 3.93 | 0.75 | 3.76 | 2.48 | 5.53 | 3.51 | 4.42 | C,B>A | 12 | 4.02 | 0.47 | 4.02 | 3.15 | 4.68 | 3.82 | 4.32 | C,B>A | 31 | 3.97 | 0.65 | 4 | 2.48 | 5.53 | 3.55 | 4.38 | C,B>A |
|  |  | ≥2 (C) | 22 | 4.04 | 0.62 | 4.14 | 2.44 | 5.6 | 3.62 | 4.38 |  | 10 | 4.14 | 0.54 | 4.11 | 3.32 | 4.98 | 3.8 | 4.46 |  | 32 | 4.07 | 0.58 | 4.14 | 2.44 | 5.6 | 3.68 | 4.39 |  |
|  | Uterus- Width [cm] | 0 (A) | 411 | 4.52 | 0.68 | 4.52 | 2.21 | 6.89 | 4.08 | 4.92 | p<0.001* | 174 | 4.77 | 0.69 | 4.76 | 2.71 | 6.93 | 4.3 | 5.18 | p=0.009* | 585 | 4.59 | 0.69 | 4.57 | 2.21 | 6.93 | 4.16 | 5.02 | p<0.001 * |
|  |  | 1 (B) | 19 | 4.96 | 0.72 | 4.78 | 3.72 | 6.75 | 4.54 | 5.49 | C,B>A | 12 | 5.29 | 0.67 | 5.14 | 4.44 | 6.48 | 4.86 | 5.71 | C,B>A | 31 | 5.09 | 0.7 | 4.95 | 3.72 | 6.75 | 4.56 | 5.69 | C,B>A |
|  |  | ≥2 (C) | 22 | 5.02 | 0.85 | 5.18 | 1.95 | 6.2 | 4.73 | 5.47 |  | 10 | 5.16 | 0.54 | 5.36 | 4.29 | 5.9 | 4.78 | 5.51 |  | 32 | 5.07 | 0.76 | 5.28 | 1.95 | 6.2 | 4.72 | 5.5 |  |
|  | Uterus- volume [ml] | 0 (A) | 411 | 34.8 | 15.46 | 31.88 | 6.04 | 120.39 | 24.36 | 42.22 | p<0.001* | 174 | 40.67 | 16.07 | 38.05 | 5.44 | 117.82 | 29.39 | 47.66 | p<0.001* | 585 | 36.55 | 15.86 | 33.72 | 5.44 | 120.39 | 25.79 | 43.59 | p<0.001 * |
|  |  | 1 (B) | 19 | 51.35 | 24.34 | 47.39 | 19.97 | 118.62 | 37.19 | 67.08 | C,B>A | 12 | 57.36 | 15.39 | 61.17 | 28.65 | 76.02 | 54.27 | 65.51 | B,C>A | 31 | 53.68 | 21.24 | 56.93 | 19.97 | 118.62 | 37.54 | 67.08 | B,C>A |
|  |  | ≥2 (C) | 22 | 56.66 | 18.78 | 50.73 | 33.58 | 100.61 | 41.43 | 72.31 |  | 10 | 55.89 | 18.07 | 58.08 | 33.58 | 89.67 | 40.06 | 67.76 |  | 32 | 56.42 | 18.27 | 51.48 | 33.58 | 100.61 | 40.28 | 69.48 |  |
| Dysmenorrhea^b^ | Endometrium- Thickness [cm] | No | 273 | 0.72 | 0.36 | 0.66 | 0.1 | 2.1 | 0.45 | 0.91 | p=0.171 | 110 | 0.86 | 0.33 | 0.9 | 0.15 | 1.8 | 0.57 | 1.04 | p=0.835 | 383 | 0.76 | 0.36 | 0.71 | 0.1 | 2.1 | 0.48 | 0.98 | p=0.168 |
|  |  | Yes | 179 | 0.74 | 0.3 | 0.7 | 0.1 | 1.68 | 0.54 | 0.95 |  | 86 | 0.86 | 0.32 | 0.86 | 0.15 | 1.63 | 0.66 | 1.06 |  | 265 | 0.78 | 0.31 | 0.76 | 0.1 | 1.68 | 0.56 | 1.01 |  |
|  | Endometrium- volume [ml] | No | 273 | 2.95 | 2.5 | 2.11 | 0.12 | 16.28 | 1.3 | 3.82 | p=0.171 | 110 | 3.84 | 2.28 | 3.42 | 0.22 | 11.2 | 1.96 | 5.17 | p=0.868 | 383 | 3.2 | 2.47 | 2.37 | 0.12 | 16.28 | 1.54 | 4.38 | p=0.219 |
|  |  | Yes | 179 | 2.97 | 2 | 2.55 | 0.26 | 13.12 | 1.5 | 4 |  | 86 | 3.79 | 2.37 | 3.41 | 0.5 | 13.39 | 2.23 | 4.78 |  | 265 | 3.24 | 2.16 | 2.83 | 0.26 | 13.39 | 1.65 | 4.3 |  |
|  | Uterus- Length [cm] | No | 273 | 4.32 | 0.78 | 4.23 | 1.87 | 6.94 | 3.84 | 4.77 | p=0.73 | 110 | 4.61 | 0.69 | 4.52 | 3.31 | 6.19 | 4.07 | 5.11 | p=0.03* | 383 | 4.41 | 0.76 | 4.32 | 1.87 | 6.94 | 3.9 | 4.92 | p=0.21 |
|  |  | Yes | 179 | 4.29 | 0.67 | 4.26 | 2.62 | 6.19 | 3.8 | 4.67 |  | 86 | 4.37 | 0.55 | 4.36 | 2.69 | 5.7 | 4.09 | 4.6 |  | 265 | 4.32 | 0.63 | 4.28 | 2.62 | 6.19 | 3.92 | 4.64 |  |
|  | Uterus- Height [cm] | No | 273 | 3.39 | 0.71 | 3.39 | 1.75 | 5.63 | 2.85 | 3.79 | p=0.342 | 110 | 3.72 | 0.64 | 3.63 | 2.16 | 5.76 | 3.29 | 4.08 | p=0.012* | 383 | 3.48 | 0.7 | 3.48 | 1.75 | 5.76 | 2.96 | 3.91 | p=0.043 * |
|  |  | Yes | 179 | 3.33 | 0.57 | 3.24 | 2.09 | 5.16 | 2.91 | 3.64 |  | 86 | 3.47 | 0.59 | 3.46 | 1.07 | 4.92 | 3.11 | 3.81 |  | 265 | 3.38 | 0.58 | 3.3 | 1.07 | 5.16 | 2.98 | 3.7 |  |
|  | Uterus- Width [cm] | No | 273 | 4.55 | 0.76 | 4.51 | 1.95 | 6.89 | 4.05 | 5.02 | p=0.277 | 110 | 4.83 | 0.73 | 4.73 | 2.71 | 6.93 | 4.32 | 5.32 | p=0.902 | 383 | 4.63 | 0.76 | 4.57 | 1.95 | 6.93 | 4.14 | 5.08 | p=0.324 |
|  |  | Yes | 179 | 4.59 | 0.59 | 4.62 | 2.21 | 6.34 | 4.22 | 4.96 |  | 86 | 4.8 | 0.65 | 4.82 | 3 | 6.75 | 4.29 | 5.22 |  | 265 | 4.66 | 0.62 | 4.66 | 2.21 | 6.75 | 4.24 | 5.06 |  |
|  | Uterus- volume [ml] | No | 273 | 37.29 | 18.8 | 33.77 | 6.04 | 120.39 | 24.48 | 46.31 | p=0.743 | 110 | 44.72 | 18.71 | 39.91 | 5.44 | 117.82 | 29.43 | 59.7 | p=0.119 | 383 | 39.42 | 19.05 | 36.12 | 5.44 | 120.39 | 26.09 | 48.27 | p=0.344 |
|  |  | Yes | 179 | 35.46 | 13.78 | 32.39 | 12.51 | 90.84 | 25.92 | 42.31 |  | 86 | 39.59 | 13.65 | 37.91 | 13.8 | 84.53 | 30.51 | 45.91 |  | 265 | 36.8 | 13.85 | 34.46 | 12.51 | 90.84 | 26.47 | 43.93 |  |
| Abnormal Uterine Bleeding^a^ | Endometrium- Thickness [cm] | No (A) | 321 | 0.71 | 0.35 | 0.65 | 0.1 | 2.1 | 0.46 | 0.9 | p=0.024* | 123 | 0.86 | 0.33 | 0.9 | 0.15 | 1.8 | 0.59 | 1.04 | p=0.867 | 444 | 0.75 | 0.35 | 0.7 | 0.1 | 2.1 | 0.48 | 0.98 | p=0.029 * |
|  |  | HMB (B) | 115 | 0.78 | 0.31 | 0.73 | 0.1 | 1.8 | 0.58 | 1.02 | B>A | 63 | 0.87 | 0.32 | 0.86 | 0.15 | 1.6 | 0.68 | 1.06 |  | 178 | 0.81 | 0.32 | 0.79 | 0.1 | 1.8 | 0.6 | 1.03 | B>A |
|  |  | IMB (C) | 16 | 0.78 | 0.24 | 0.81 | 0.3 | 1.14 | 0.57 | 0.92 |  | 10 | 0.81 | 0.35 | 0.86 | 0.27 | 1.39 | 0.54 | 1 |  | 26 | 0.79 | 0.29 | 0.82 | 0.27 | 1.39 | 0.56 | 1 |  |
|  | Endometrium- volume [ml] | No (A) | 321 | 2.87 | 2.38 | 2.12 | 0.12 | 16.28 | 1.27 | 3.8 | p=0.062 | 123 | 3.79 | 2.13 | 3.6 | 0.22 | 11.2 | 2.06 | 5.11 | p=0.872 | 444 | 3.13 | 2.35 | 2.4 | 0.12 | 16.28 | 1.47 | 4.27 | p=0.133 |
|  |  | HMB (B) | 115 | 3.18 | 2.21 | 2.57 | 0.4 | 12.56 | 1.71 | 4.08 |  | 63 | 3.82 | 2.47 | 3.38 | 0.5 | 13.39 | 2.2 | 4.66 |  | 178 | 3.41 | 2.32 | 2.83 | 0.4 | 13.39 | 1.81 | 4.48 |  |
|  |  | IMB (C) | 16 | 3.12 | 1.57 | 2.58 | 1.27 | 6.35 | 2.07 | 4.42 |  | 10 | 4.01 | 3.45 | 3.06 | 0.78 | 12.05 | 1.54 | 5.23 |  | 26 | 3.46 | 2.44 | 2.74 | 0.78 | 12.05 | 1.69 | 4.6 |  |
|  | Uterus- Length [cm] | No (A) | 321 | 4.31 | 0.76 | 4.23 | 1.87 | 6.94 | 3.82 | 4.75 | p=0.973 | 123 | 4.54 | 0.66 | 4.5 | 3.23 | 6.19 | 4.08 | 5.06 | p=0.541 | 444 | 4.37 | 0.74 | 4.31 | 1.87 | 6.94 | 3.87 | 4.82 | p=0.975 |
|  |  | HMB (B) | 115 | 4.33 | 0.66 | 4.25 | 2.74 | 6.1 | 3.9 | 4.69 |  | 63 | 4.42 | 0.59 | 4.38 | 2.69 | 5.81 | 4.08 | 4.88 |  | 178 | 4.36 | 0.63 | 4.28 | 2.69 | 6.1 | 3.98 | 4.7 |  |
|  |  | IMB (C) | 16 | 4.35 | 0.72 | 4.27 | 3.28 | 5.88 | 3.98 | 4.54 |  | 10 | 4.5 | 0.71 | 4.39 | 3.51 | 5.95 | 4.19 | 4.55 |  | 26 | 4.41 | 0.71 | 4.38 | 3.28 | 5.95 | 4.05 | 4.55 |  |
|  | Uterus- Height [cm] | No (A) | 321 | 3.33 | 0.67 | 3.29 | 1.75 | 5.63 | 2.84 | 3.71 | p=0.366 | 123 | 3.65 | 0.63 | 3.57 | 2.16 | 5.76 | 3.18 | 4.03 | p=0.749 | 444 | 3.42 | 0.67 | 3.41 | 1.75 | 5.76 | 2.91 | 3.8 | p=0.562 |
|  |  | HMB (B) | 115 | 3.44 | 0.63 | 3.36 | 2.41 | 5.6 | 3 | 3.76 |  | 63 | 3.54 | 0.64 | 3.51 | 1.07 | 5.06 | 3.17 | 3.84 |  | 178 | 3.47 | 0.63 | 3.43 | 1.07 | 5.6 | 3.06 | 3.84 |  |
|  |  | IMB (C) | 16 | 3.43 | 0.64 | 3.23 | 2.7 | 4.96 | 3.01 | 3.62 |  | 10 | 3.59 | 0.53 | 3.38 | 2.83 | 4.61 | 3.27 | 3.98 |  | 26 | 3.49 | 0.59 | 3.3 | 2.7 | 4.96 | 3.09 | 3.88 |  |
|  | Uterus- Width [cm] | No (A) | 321 | 4.53 | 0.71 | 4.52 | 1.95 | 6.85 | 4.05 | 4.97 | p=0.076 | 123 | 4.78 | 0.69 | 4.76 | 2.71 | 6.93 | 4.31 | 5.22 | p=0.63 | 444 | 4.6 | 0.71 | 4.57 | 1.95 | 6.93 | 4.15 | 5.04 | p=0.036 * |
|  |  | HMB (B) | 115 | 4.68 | 0.68 | 4.67 | 2.21 | 6.89 | 4.25 | 5.05 |  | 63 | 4.89 | 0.72 | 4.9 | 3 | 6.75 | 4.32 | 5.42 |  | 178 | 4.75 | 0.7 | 4.72 | 2.21 | 6.89 | 4.28 | 5.13 | B>A |
|  |  | IMB (C) | 16 | 4.41 | 0.56 | 4.52 | 3.3 | 5.38 | 4.13 | 4.72 |  | 10 | 4.83 | 0.64 | 4.55 | 4.16 | 6.03 | 4.34 | 5.2 |  | 26 | 4.57 | 0.62 | 4.52 | 3.3 | 6.03 | 4.24 | 4.89 |  |
|  | Uterus- volume [ml] | No (A) | 321 | 36.03 | 17.13 | 32.69 | 6.04 | 120.39 | 24.18 | 43.41 | p=0.315 | 123 | 43.03 | 16.98 | 39.25 | 10.26 | 117.82 | 30.2 | 56.87 | p=0.659 | 444 | 37.97 | 17.35 | 34.44 | 6.04 | 120.39 | 25.77 | 46.61 | p=0.292 |
|  |  | HMB (B) | 115 | 38.15 | 16.92 | 35.66 | 12.51 | 118.62 | 27.65 | 45.62 |  | 63 | 42.12 | 16.18 | 39.8 | 13.8 | 89.67 | 31.34 | 47.48 |  | 178 | 39.55 | 16.72 | 37.44 | 12.51 | 118.62 | 28.57 | 46.31 |  |
|  |  | IMB (C) | 16 | 35.79 | 15.19 | 31.41 | 19.72 | 70.25 | 25.86 | 41.42 |  | 10 | 37.77 | 19.93 | 31.53 | 5.44 | 78.37 | 29.22 | 46.8 |  | 26 | 36.55 | 16.8 | 31.41 | 5.44 | 78.37 | 26.37 | 42.88 |  |
| Polycystic Ovarian Morphology^b^ | Endometrium- Thickness [cm] | No | 122 | 0.8 | 0.36 | 0.76 | 0.16 | 1.96 | 0.52 | 1.02 | p=0.015* | 115 | 0.89 | 0.32 | 0.9 | 0.28 | 1.8 | 0.68 | 1.06 | p=0.156 | 237 | 0.84 | 0.35 | 0.85 | 0.16 | 1.96 | 0.57 | 1.04 | p<0.001 * |
|  |  | Yes | 330 | 0.7 | 0.32 | 0.66 | 0.1 | 2.1 | 0.47 | 0.88 |  | 81 | 0.82 | 0.32 | 0.85 | 0.15 | 1.75 | 0.58 | 1.02 |  | 411 | 0.73 | 0.33 | 0.68 | 0.1 | 2.1 | 0.49 | 0.93 |  |
|  | Endometrium- volume [ml] | No | 122 | 3.34 | 2.4 | 2.76 | 0.13 | 14.64 | 1.56 | 4.28 | p=0.005* | 115 | 3.86 | 2.31 | 3.45 | 0.59 | 13.39 | 2.18 | 4.76 | p=0.727 | 237 | 3.59 | 2.36 | 3.13 | 0.13 | 14.64 | 1.94 | 4.62 | p<0.001 * |
|  |  | Yes | 330 | 2.82 | 2.27 | 2.08 | 0.12 | 16.28 | 1.29 | 3.78 |  | 81 | 3.75 | 2.33 | 3.36 | 0.22 | 11.2 | 1.81 | 5.09 |  | 411 | 3 | 2.31 | 2.22 | 0.12 | 16.28 | 1.38 | 4.04 |  |
|  | Uterus- Length [cm] | No | 122 | 4.47 | 0.83 | 4.35 | 1.87 | 6.94 | 3.95 | 4.97 | p=0.013* | 115 | 4.53 | 0.66 | 4.4 | 2.69 | 6.16 | 4.11 | 5.04 | p=0.573 | 237 | 4.5 | 0.75 | 4.4 | 1.87 | 6.94 | 4.03 | 5.01 | p=0.001 * |
|  |  | Yes | 330 | 4.25 | 0.69 | 4.21 | 2.11 | 6.55 | 3.8 | 4.68 |  | 81 | 4.47 | 0.62 | 4.44 | 3.31 | 6.19 | 4.08 | 4.89 |  | 411 | 4.3 | 0.68 | 4.25 | 2.11 | 6.55 | 3.84 | 4.71 |  |
|  | Uterus- Height [cm] | No | 122 | 3.53 | 0.69 | 3.45 | 2.11 | 5.6 | 3.08 | 3.99 | p=0.001* | 115 | 3.71 | 0.6 | 3.62 | 2.37 | 5.1 | 3.27 | 4.07 | p=0.024 * | 237 | 3.62 | 0.65 | 3.52 | 2.11 | 5.6 | 3.18 | 4.03 | p<0.001 * |
|  |  | Yes | 330 | 3.3 | 0.64 | 3.24 | 1.75 | 5.63 | 2.85 | 3.65 |  | 81 | 3.47 | 0.65 | 3.51 | 1.07 | 5.76 | 3.08 | 3.84 |  | 411 | 3.34 | 0.64 | 3.33 | 1.07 | 5.76 | 2.88 | 3.68 |  |
|  | Uterus- Width [cm] | No | 122 | 4.66 | 0.71 | 4.66 | 1.95 | 6.89 | 4.22 | 5.1 | p=0.048* | 115 | 4.94 | 0.68 | 4.94 | 3.46 | 6.93 | 4.44 | 5.41 | p=0.003 * | 237 | 4.8 | 0.71 | 4.74 | 1.95 | 6.93 | 4.31 | 5.27 | p<0.001 * |
|  |  | Yes | 330 | 4.52 | 0.69 | 4.52 | 2.21 | 6.85 | 4.08 | 4.95 |  | 81 | 4.65 | 0.68 | 4.6 | 2.71 | 6.31 | 4.25 | 4.99 |  | 411 | 4.55 | 0.69 | 4.53 | 2.21 | 6.85 | 4.13 | 4.96 |  |
|  | Uterus- volume [ml] | No | 122 | 40.92 | 18.81 | 36.83 | 7.51 | 100.61 | 28.69 | 49.62 | p=0.001* | 115 | 44.4 | 16.99 | 40.01 | 5.44 | 89.67 | 30.76 | 57.7 | p=0.045 * | 237 | 42.61 | 17.99 | 38.64 | 5.44 | 100.61 | 29.88 | 53.47 | p<0.001 * |
|  |  | Yes | 330 | 34.95 | 16.01 | 31.76 | 6.04 | 120.39 | 24.3 | 42.31 |  | 81 | 39.74 | 16.33 | 37.48 | 10.26 | 117.82 | 29.31 | 46.3 |  | 411 | 35.89 | 16.17 | 32.72 | 6.04 | 120.39 | 25.22 | 43.48 |  |
| Dominant Follicle^b^ | Endometrium- Thickness [cm] | No | 363 | 0.7 | 0.35 | 0.65 | 0.1 | 2.1 | 0.44 | 0.9 | p<0.001* | 146 | 0.85 | 0.34 | 0.88 | 0.15 | 1.8 | 0.6 | 1.04 | p=0.254 | 509 | 0.74 | 0.35 | 0.7 | 0.1 | 2.1 | 0.48 | 0.97 | p<0.001 * |
|  |  | Yes | 89 | 0.83 | 0.27 | 0.81 | 0.23 | 1.69 | 0.64 | 1.02 |  | 50 | 0.9 | 0.28 | 0.92 | 0.4 | 1.51 | 0.66 | 1.08 |  | 139 | 0.86 | 0.28 | 0.85 | 0.23 | 1.69 | 0.64 | 1.04 |  |
|  | Endometrium- volume [ml] | No | 363 | 2.84 | 2.41 | 2.04 | 0.12 | 16.28 | 1.27 | 3.74 | p<0.001* | 146 | 3.72 | 2.32 | 3.25 | 0.22 | 13.39 | 1.96 | 4.82 | p=0.233 | 509 | 3.09 | 2.42 | 2.34 | 0.12 | 16.28 | 1.42 | 4.15 | p<0.001 * |
|  |  | Yes | 89 | 3.44 | 1.77 | 3.08 | 0.26 | 8.87 | 2.11 | 4.4 |  | 50 | 4.11 | 2.29 | 3.79 | 1.12 | 12.05 | 2.18 | 5.33 |  | 139 | 3.68 | 1.99 | 3.34 | 0.26 | 12.05 | 2.18 | 4.68 |  |
|  | Uterus- Length [cm] | No | 363 | 4.29 | 0.76 | 4.2 | 1.87 | 6.94 | 3.82 | 4.74 | p=0.077 | 146 | 4.47 | 0.61 | 4.42 | 2.69 | 6.19 | 4.08 | 4.86 | p=0.324 | 509 | 4.34 | 0.72 | 4.28 | 1.87 | 6.94 | 3.87 | 4.78 | p=0.032 * |
|  |  | Yes | 89 | 4.41 | 0.63 | 4.41 | 2.88 | 6.1 | 4.01 | 4.74 |  | 50 | 4.6 | 0.72 | 4.44 | 3.31 | 6.16 | 4.08 | 5.21 |  | 139 | 4.48 | 0.66 | 4.41 | 2.88 | 6.16 | 4.03 | 4.96 |  |
|  | Uterus- Height [cm] | No | 363 | 3.33 | 0.68 | 3.28 | 1.75 | 5.63 | 2.84 | 3.67 | p=0.007* | 146 | 3.59 | 0.65 | 3.51 | 1.07 | 5.76 | 3.16 | 3.93 | p=0.241 | 509 | 3.4 | 0.68 | 3.36 | 1.07 | 5.76 | 2.9 | 3.77 | p=0.002 * |
|  |  | Yes | 89 | 3.5 | 0.52 | 3.42 | 2.51 | 4.74 | 3.1 | 3.84 |  | 50 | 3.68 | 0.56 | 3.65 | 2.53 | 4.98 | 3.34 | 4.06 |  | 139 | 3.56 | 0.54 | 3.49 | 2.51 | 4.98 | 3.17 | 3.95 |  |
|  | Uterus- Width [cm] | No | 363 | 4.52 | 0.72 | 4.52 | 1.95 | 6.85 | 4.08 | 4.94 | p=0.011* | 146 | 4.77 | 0.7 | 4.68 | 2.71 | 6.93 | 4.3 | 5.22 | p=0.07 | 509 | 4.6 | 0.72 | 4.57 | 1.95 | 6.93 | 4.15 | 5.02 | p=0.001 * |
|  |  | Yes | 89 | 4.72 | 0.61 | 4.7 | 2.99 | 6.89 | 4.39 | 5.06 |  | 50 | 4.96 | 0.66 | 5 | 3.75 | 6.75 | 4.46 | 5.43 |  | 139 | 4.8 | 0.64 | 4.8 | 2.99 | 6.89 | 4.4 | 5.16 |  |
|  | Uterus- volume [ml] | No | 363 | 35.94 | 17.69 | 32.15 | 6.04 | 120.39 | 23.85 | 43.18 | p=0.005* | 146 | 41.74 | 16.25 | 38.13 | 10.26 | 117.82 | 29.99 | 49.8 | p=0.32 | 509 | 37.6 | 17.47 | 34.01 | 6.04 | 120.39 | 25.56 | 45.73 | p=0.003 * |
|  |  | Yes | 89 | 39.11 | 13.62 | 37.23 | 14.1 | 92.08 | 30.36 | 46.64 |  | 50 | 44.62 | 18.46 | 40.06 | 5.44 | 89.67 | 30.36 | 59.49 |  | 139 | 41.09 | 15.69 | 38.35 | 5.44 | 92.08 | 30.33 | 49.28 |  |

^a^ p - Kruskal-Wallis test + post-hoc analysis (Dunn test), SD - standard deviation, Q1 - lower quartile, Q3 - upper quartile, * statistically significant (p<0.05), ^b^ p - Mann-Whitney test, SD - standard deviation, Q1 - lower quartile, Q3 - upper quartile
